# Whole Dietary Patterns, Cognitive Decline and Cognitive Disorders: A Systematic Review of Prospective and Intervention Studies

**DOI:** 10.3390/nu15020333

**Published:** 2023-01-09

**Authors:** Rebecca F. Townsend, Danielle Logan, Roisin F. O’Neill, Federica Prinelli, Jayne V. Woodside, Claire T. McEvoy

**Affiliations:** 1 Centre for Public Health, Queen’s University Belfast, Belfast BT12 6BA, UK; 2 Epidemiology Unit, Institute of Biomedical Technologies, National Research Council, 93 20054 Milan, Italy; 3 Institute for Global Food Security, Queen’s University Belfast, Belfast BT9 5DL, UK

**Keywords:** dietary patterns, cognition, dementia, cognitive disorders

## Abstract

Dementia prevalence is a global public health concern. Adherence towards a healthy dietary pattern (DP) may reduce the risk of cognitive decline and dementia. This narrative systematic review aimed to synthesise prospective and intervention study data to evaluate the impact of *a*-posteriori and *a*-priori derived DPs on cognitive ageing, from cognitive decline to incident dementia. Ninety-three studies were included: 83 prospective studies and 10 randomised controlled trials (RCT). Most prospective studies (77%) examined *a*-priori DPs, with the Mediterranean diet examined most frequently. A total of 52% of prospective and 50% of RCTs reported a protective relationship between ‘healthy’ DPs and global cognitive decline. Overall, 59% of prospective studies reported positive associations between healthy DPs and risk of cognitive disorder. Incident cognitive disorder was examined by only one intervention study (subgroup analysis) which reported a beneficial effect of a low-fat diet on risk of probable dementia in women. Unhealthy DPs were examined less frequently (*n* = 17; 21%), with 41% of these studies reporting associations between adherence and poorer cognitive outcomes. Overall, there were mixed results for healthy and unhealthy DPs on cognition, likely due to between-study heterogeneity. Standardisation of diet exposure and cognitive outcome measurement would help to reduce this. Future research would benefit from investigating effects of culturally appropriate DPs on individual cognitive domains and incident cognitive disorders in diverse and high-risk populations.

## 1. Introduction

The global challenges associated with the expansion of an ageing population are well documented. One of significant importance is the increasing prevalence of dementia, which is projected to increase from 50 million to 152 million by 2050, for which there is a lack of pharmacological treatment. However, up to 40% of future dementia cases could be potentially prevented or delayed by targeting modifiable factors [1]. Dietary modification has been considered as a potential strategy to reduce population incidence of dementia via indirect and direct mechanisms [2]. In previous years, the diet-dementia field has shifted away from exploring the role of single nutrients towards the study of dietary patterns (DPs) [3]. The rationale for this shift to DPs is two-fold; firstly, DPs may provide a better reflection of individuals’ eating behaviours compared to the study of single nutrients or foods [4]. Secondly, studying a DP also accounts for the synergies between nutrients and food components, which may exert stronger effects on cognition compared to single nutrients alone [5]. Hence, a DP could be considered healthy or unhealthy depending on the combination of foods consumed by the individual [4]. Healthy DPs generally emphasise high intakes of fruit, vegetables and wholegrains, whereas unhealthy DPs typically are higher in foods considered unhealthy such as, confectionary, meat and processed foods. There are different methods to analyse DPs, with the most common approaches applying ‘*a*-posteriori’ methods to derive data driven DPs from the population or ‘*a*-priori’ dietary scores that are developed using existing evidence for diet and health outcomes.

Observational studies which explored associations between healthy DPs and cognitive decline displayed mixed results [6,7,8,9,10,11]. Additionally, few intervention studies have been conducted to investigate the effects of DP adherence on cognitive decline. Most of those which do exist investigate the effects of cognitive outcomes within secondary analyses and are often short in duration. The limited evidence from such studies has also been inconsistent in confirming that greater adherence to a healthy DP confers neuroprotection [12,13,14].

Several systematic reviews have attempted to synthesise findings in the field of DPs and cognitive ageing but have been limited in scope [15,16,17,18,19,20,21,22,23,24,25,26]. Some reviews have focused exclusively on healthy DPs considered to be cardioprotective, such as the Mediterranean or DASH diets [16,25]. Although this provides insight into the impact of such DPs on cognition, they neglect other DPs which may be consumed by western populations. This includes *a*-posteriori derived DPs termed “western”, or those recommended by government and public health organizations, such as the Healthy Eating Index (HEI)-2005. Other reviews have included a range of DPs, but limited searches to either outcome of cognitive function or decline [22,27], or the diagnosis of cognitive disorders such as mild cognitive impairment (MCI), incident all-cause dementia or dementia subtypes, for example, Alzheimer’s disease (AD) [20,21,23,26]. Some reviews have also restricted populations using age limits, i.e., only included middle-age populations [18,24] or populations aged ≥50 years [15,17,19]. It is proposed a healthy DP likely confers small but cumulative effects on cognition, prior to the onset of any incident cognitive disorder (often occurring during later-life) [18,28]. However, the optimal duration of DP adherence to reap cognitive benefits remains unknown [3]. Although it may coincide with the onset of cognitive ageing (approx. 30 years of age), adhering to a healthy DP at the onset of adulthood (≥18 years), may provide the greatest neuroprotection [29]. Consequently, it is important to consider studies following this time point, and a broad array of potentially relevant outcomes to capture any changes in cognition (e.g., cognitive decline, diagnosis of incident cognitive disorder). Most prior reviews also included cross-sectional studies, thus making it difficult to draw causal associations due to concurrent measurement of exposures and outcomes [15,16,18,19,20,22].

One previous systematic review [17], synthesised evidence from prospective and intervention studies published up to 2017. Inclusion criteria considered a broad range of DPs in relation to all relevant outcomes of cognitive ageing. From 38 studies, the review found that most evidence (17/23 studies) supported a role for Mediterranean diet in promoting cognitive health. It also found limited but promising evidence for the beneficial impacts of other healthy DPs (e.g., DASH (2/3 studies) and anti-inflammatory DP (3/3 studies). A substantial number of studies have been published in the diet-dementia field since then which have yet to be comprehensively synthesized.

### Review Aims

This systematic review aimed to address the following questions.

i. What are the associations between any *a*-priori or *a*-posteriori DP and outcomes of cognitive ageing across the adult life course?
ii. What is the effect of DPs on cognitive ageing outcomes?

## 2. Methods

### 2.1. Study Protocol

This systematic review evaluated prospective studies and randomised control trials (RCT)) which investigated any whole DP in relation to outcomes of cognitive decline and any mild or major cognitive disorder, complementing neuroimaging outcomes which have already been published [30]. The review protocol was registered with PROSPERO (CRD42020181423) and undertaken in accordance with the Centre for Reviews and Dissemination guidance for systematic reviews in healthcare.

### 2.2. Inclusion and Exclusion Criteria

The review included only prospective or RCT study design and eligible studies met the following criteria; (i) assessed adherence to ≥1 whole DP (ii) provided ≥1 relevant cognitive outcome and (iii) evaluated the association/effect of DP adherence on cognitive outcomes. Within the context of this review, relevant cognitive outcomes were classified as 1. change in cognitive performance where measured cognitive function was repeated at two time points; 2. cognitive decline where measured cognitive function was repeated ≥3 times; 3. MCI [31]; and 4. incident major cognitive disorder, e.g., dementia and dementia subtypes [31]. Studies that focused on nutrients alone, macronutrient groups, single foods, calorie restriction or weight loss without consideration of overall diet quality were excluded. Studies involving multi-domain interventions or exposures were also excluded due to difficulty in extracting the effect of diet alone.

### 2.3. Search Strategy and Data Sources

A detailed search strategy was conducted using two major databases (Ovid MEDLINE and EMBASE). The first search was undertaken on 5th March 2020 and updated using steps outlined by Bramer and Bain [32] to capture studies published to 2nd August 2022. A search strategy was developed using key terms associated with DPs and cognitive outcomes and is provided in Table S1. Searches were limited to studies of human adults (≥18 years of age) and published in English.

### 2.4. Data Selection and Extraction

Study selection and extraction were managed using Endnote software (version X9) and Rayyan online software. Title screening was completed (R.F.T) and articles without keywords relating to the review were excluded. The abstract screening was completed by two independent reviewers (R.F.T & C.ME/D.L), followed by a review of full texts to assess the eligibility of remaining articles. If required, disagreements were resolved via discussion with a third member of the research team (J.V.W). To avoid duplication of eligible studies drawn from the same cohort those with the longest-follow up period were included and the remaining studies were excluded unless they reported different exposures or outcomes of interest.

Data extraction was completed using a standardised structured table. For prospective studies this included: authors, year of publication, study cohort name (if applicable) and country; study characteristics including follow-up period (years), sample size and mean age at baseline; method to derive DP (*a*-priori or *a*-posteriori); dietary assessment methodology and time points; DP(s) derived (scoring reference if *a*-priori), cognitive outcomes including methodology and assessment time points; primary outcomes of interest and summary of main findings from the primary outcome, including covariates included in fully adjusted models (where applicable). For intervention studies, information collected included: intervention design, duration and follow-up (if applicable); randomisation method; sample size; population characteristics; intervention description; diet assessment methods used, and DPs assessed; primary outcomes and summary of study findings in relation to primary outcomes.

In relation to outcomes of cognitive performance/cognitive decline, we extracted measures or summed scores for global cognition, as this outcome was reported most frequently across the studies.

### 2.5. Risk of Bias Assessment

Study quality was assessed by two independent reviewers (R.F.T & R.F.ON/D.L) for prospective studies and intervention studies (R.F.T & C.ME/D.L). The Newcastle Ottawa Scale (N.O.S) was used to evaluate the quality of prospective studies [33]. Up to 9 stars in total could be awarded (4 for study selection; 2 for comparability; 3 for outcome measurement). In relation to the outcome domain, all studies were provided with a star for assessment of the outcome, due to the nature of cognitive assessment which is typically conducted by a trained individual as standard. Stars obtained per domain were then summed and studies were categorised as high (9 stars), medium (7–8 stars), or low quality (≤6 stars). For intervention studies, the Cochrane RoB-2 tool was used [34]. The tool assessed bias within six domains (selection, performance, detection, attrition, reporting and other bias), an algorithm was then used to generate a risk of bias per domain and overall categorisation as low risk, some concerns, or high risk [34].

### 2.6. Data Synthesis

Due to the considerable between-study heterogeneity found in relation to reporting of data and statistical analyses, a meta-analysis was not possible and instead a rigorous narrative approach was used to present results. For each DP and cognitive outcome, we summarized the findings as the proportion of studies reporting a positive (beneficial association), null (no association) and negative (adverse association).

## 3. Results

### 3.1. Study Selection

**Figure 1:**
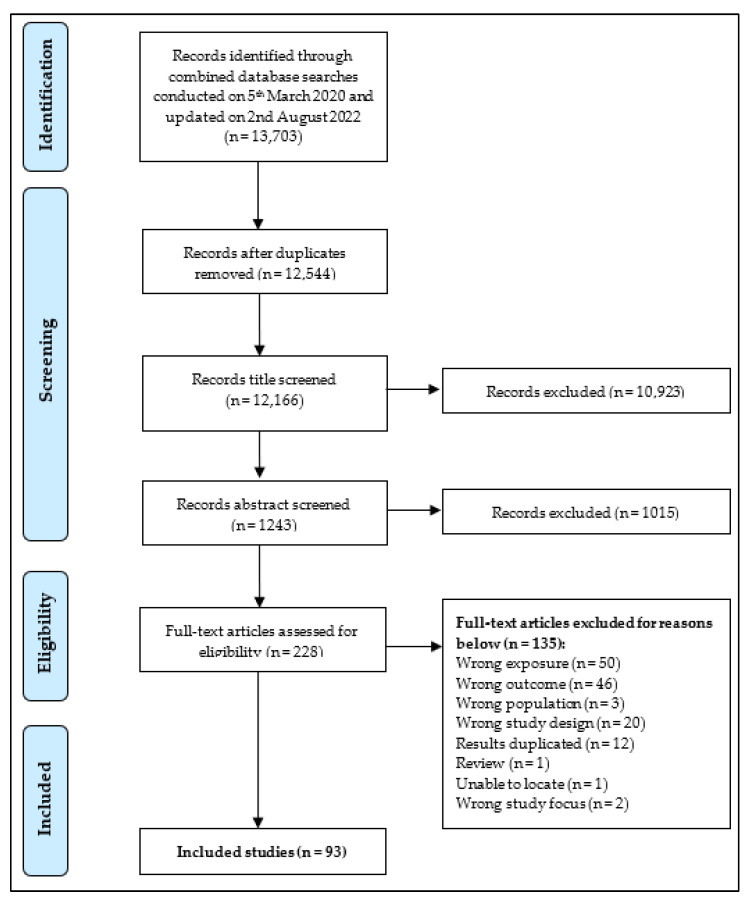
Preferred reporting items for systematic reviews and meta-analyses (PRISMA) flow diagram showing the selection process for studies included in review.

The preferred reporting items for systematic reviews and meta-analyses (PRISMA) flow diagram for eligible studies is summarised in Figure 1. In brief, the literature searches yielded 13,703 titles of which 1159 were duplicates and excluded. Of the remaining titles, 12,256 were excluded after abstract screening. Full text of 228 articles were independently reviewed and 135 of these did not meet the inclusion criteria for reasons outlined in Figure 1. A total of 93 studies (83 prospective studies and 10 randomised control trials (RCT)) were eligible for inclusion. All included studies were published in the last 16 years.

### 3.2. Overview of Prospective Studies

From the 83 prospective studies, 18 [7,11,35,36,37,38,39,40,41,42,43,44,45,46,47,48,49,50] assessed change in global cognitive performance (total *n* = 30,932), 44 [6,8,9,10,51,52,53,54,55,56,57,58,59,60,61,62,63,64,65,66,67,68,69,70,71,72,73,74,75,76,77,78,79,80,81,82,83,84,85,86,87,88,89,90] assessed global cognitive decline (total *n* = 190,572) and 28 studies assessed risk of cognitive disorders (*n* = 220,400 max.) which included risk of MCI (7 studies) [11,91,92,93,94,95,96], risk of all-cause incident dementia (17 studies) [87,97,98,99,100], risk of AD (7 studies) [60,76,101,102,103,104,105] and other outcomes related to risk of cognitive disorder (3 studies) [91,92,105]. The sample size ranged from 70 to 37,689, and the length of follow-up ranged from 1 to 27 years. Two studies were conducted in young adulthood (35–50 years), 19 studies in middle-age (51–64 years), 38 studies in older age (65–74 years), 13 studies in later life (≥75 years) and 11 among mixed age categories. No study included exclusively younger adults (18–34 years).

Thirty-six studies conducted within the same cohort were included due to differences in reported exposures/outcomes (ARIC (*n* = 2), CACHE (*n* = 2), CHAP (*n* = 3), CHNS (*n* = 3), MAP (*n* = 4), NHS (*n* = 2), NU-AGE (*n* = 2), PATH (*n* = 2), PREDIMED-Plus (*n* = 2), REGARDS (*n* = 2), SNAC-K (*n* = 2), SUN (*n* = 2), Sydney Memory & Ageing Study (*n* = 2), WHICAP (*n* = 3), Whitehall (*n* = 2), WHS (*n* = 2), WHIMS (*n* = 2).

#### 3.2.1. Assessment of Dietary Intake in Prospective Studies

The majority (64/83; 77%) of studies used food-frequency questionnaires (FFQs) to assess dietary intake. Most (52/64; 81%), were self-administered, although 12 studies (18%) reported FFQ administration by a trained individual [7,45,48,55,63,64,76,84,92,93,106,107]. Eleven studies (12%) used other recall methods (e.g., 24 hr recalls or diet history) of which, 7 were administered by trained researchers/dietitians [43,47,60,62,72,79,98,108], 2 via computerised software [38,42] and one via weighed dietary recall [49]. Five studies (6%) used diet diaries [35,39,40,47,104] and 4 of these studies specifically stated food diaries had received input from trained individuals (i.e., quality checking or comprehensive instructional guidance) [39,40,47,104]. Screening questionnaires purposely made to ascertain adherence to a DP were used in 3 studies (3%) [67,81,84]. Three studies used >1 method to measure dietary intake [47,60,84]. Most (58/83; 70%) studies assessed dietary intake at 1-time point to derive DPs. The remaining 24 studies [10,35,38,40,41,42,43,49,51,54,66,69,75,79,81,84,93,100,102,103,107,109,110] used repeated measures of dietary intake and one study [90] lacked information about the time points for diet assessment.

#### 3.2.2. Assessment of DPs in Prospective Studies

*a*-priori DPs were examined in 64 out of 83 (77%) prospective studies. The Mediterranean diet was examined most frequently by 41 studies, followed by the DASH diet (17 studies). A total of 24 studies (30%) explored *a*-posteriori DPs, using either principal component analysis (12 studies), exploratory factor analysis (4 studies) or reduced rank regression (RRR) (7 studies) [6,46,49,70,77,79,98,102,105,106,111]. One study applied cluster analysis to derive DPs [64]. There were 5 studies that analysed both *a*-priori and *a*-posteriori DPs [10,53,61,72,73,77].

**Figure 2:**
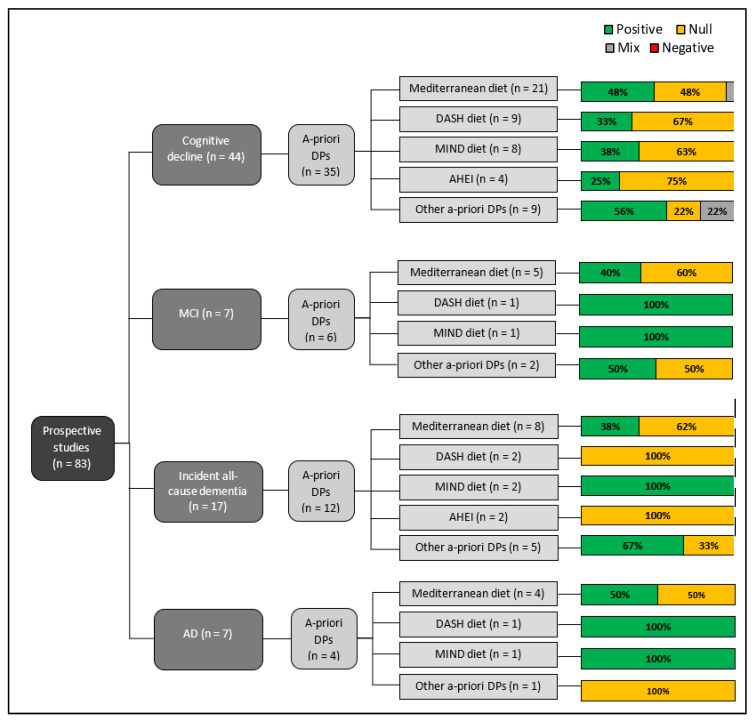
Overview of reported prospective associations between *a*-priori DPs, cognitive decline and incident cognitive disorder (% per number of included studies).

**Figure 3:**
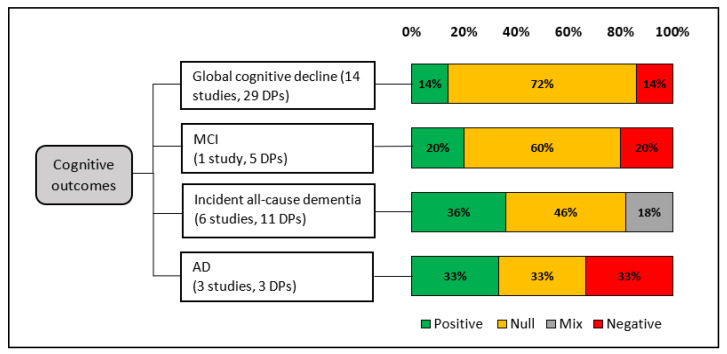
Overview of reported prospective associations between *a*-posteriori DPs and selected cognitive outcomes (% per number of included DPs).

Figure 2 and Figure 3 provide an overview of prospective associations between *a*-priori DPs (Figure 2) and *a*-posteriori DPs (Figure 3) in relation to cognitive decline and incident cognitive disorder. The *a*-priori Mediterranean DP followed by the DASH and MIND DP were investigated most frequently across the cognitive outcomes.

### 3.3. Risk of Bias in Prospective Studies

An overview of the risk of bias assessment algorithm and rating of prospective studies according to NOS is provided in Table S2. Briefly, 14 studies [7,40,43,45,47,60,62,64,76,79,92,98,101,108] were rated as high, 8 as low [51,54,67,74,80,86,99,102], and the remaining 61 as medium quality.

### 3.4. Associations between DPs and Global Cognitive Performance

In total, 18 studies examined associations between DPs (*a*-priori DPs *n* = 14; *a*-posteriori DPs *n* = 4) and cognitive performance [7,11,35,36,37,38,39,40,41,42,43,44,45,46,47,48,49,50] with follow up ranging from 1 to 17 years. An overview of these studies is provided in Table S3. Greater adherence to the Mediterranean diet was positively associated with better global cognitive performance in 5 [7,39,41,44,45] out of 9 studies (55%) [11,42,48,50]. The DASH [37,41,48] or MIND diet [43,48] were not related to global cognitive function. Other healthy *a*-priori DPs (i.e., a healthy food choice score, the modified alternative healthy eating index, and the *a*-priori dietary quality score) demonstrated some benefits for cognition, as 3 [35,36,41] out of 4 studies [35,36,40,41] reported positive associations between the diet score and global cognition. In the 4 studies that examined *a*-posteriori DPs, 2 studies [38,46] reported null findings and in the 2 remaining studies [47,49] adherence to 4 healthy DPs (termed legumes DP, plant-protein DP, beans and mushroom DP and beverages and nuts DP) were associated with better global cognitive performance.

#### 3.4.1. Associations between DPs and Global Cognitive Decline

In total, 44 prospective studies [6,8,9,10,51,52,53,54,55,56,57,58,59,60,61,62,63,64,65,66,67,68,69,70,71,72,73,74,75,76,77,78,79,80,81,82,83,84,85,86,87,88,89,90] assessed associations between DPs and cognitive decline (i.e., cognitive performance assessed at ≥2-time points), ranging from 2 to 24.8 years in follow-up length. An overview of these studies is provided in Table S4. A total of 27 studies [10,51,52,53,54,55,56,57,58,61,62,65,66,69,70,72,74,75,76,79,81,82,83,84,86,87] examined a composite measure of global cognition, and 17 used a global cognitive screener (7 studies used MMSE) [6,8,60,73,77,90], 5 studies [66,71,78,88,89] used the 3MS, 3 studies [9,63,68] used a variation of Telephone Interview for Cognitive Status (TICS), 1 study [85] used the short portable mental status questionnaire (SPMSQ) and 1 study [67] used the Preclinical Alzheimer’s Cognitive Composite Score-4 (PACC-4)). Assessment was performed at 3 time points in 17 studies [6,9,52,55,59,61,68,70,72,74,75,76,77,80,81,84,85], at ≥4 time points in 16 studies [8,56,57,58,62,63,64,69,71,73,78,82,86,88,89,90]. In the remaining 11 studies [10,51,53,54,60,65,66,67,79,83,87] there were variations in the number of time points used in the sample (i.e., ≥3, ranging up to 10). The majority (30/44 studies; 68%) assessed global cognitive decline in older adults. From 44 studies, 35 examined *a*-priori DPs and 14 [6,61,62,68,70,71,72,73,77,79] examined *a*-posteriori DPs, and 5 studies [10,61,72,73,77] examined both. The results are discussed below.

##### Mediterranean Diet and Global Cognitive Decline

From the 21 studies that investigated adherence to the Mediterranean diet, 14 studies (67%) [8,9,52,56,60,61,69,72,73,75,76,86,88,112] used a population-specific score, based on median food intake thresholds derived from Trichopoulou et al. [113] (MDS). Seven studies (33%) [57,64,65,77,82,83,86] used a Greek Mediterranean diet score based on absolute food intake targets from Panagiotakos et al. [114] (MedDiet index). Three studies used other score systems, including those from Martínez-González et al. [115] or Lopez-Garcia et al. [116]. Three studies used >1 Mediterranean diet indices [9,57,86]. Higher Mediterranean diet score was associated with slower cognitive decline in 10 out of 21 (48%) studies [8,52,64,65,69,76,77,82,83,88] with follow up ranging from 2 to 15 years. Two of these studies reported significant associations only among sub-groups of the cohort (i.e., among those with type 2 diabetes [52], or black individuals [64]. In contrast, 11 studies (48%) [9,57,60,61,63,72,73,74,75,81,86] found no association between the Mediterranean diet score and cognitive decline over 3 to 9 years. When results were analysed by score system, 71% (5/7 studies) of those using the MedDiet index from Panagiotakos et al. [114] and 64% (9/14 studies) of those using the MDS from Trichopoulou et al. [113] linked higher Mediterranean diet score with slower global cognitive decline.

##### DASH Diet and Global Cognitive Decline

Nine studies explored associations between adherence to DASH and global cognitive decline [9,52,54,57,65,77,83,84,88]. From these, 67% (6/9 studies) used indices from Fung et al. [117] or an adaptation of this index, to determine adherence [9,52,54,57,84,88]. Two studies [66,83] used the index from Folsom et al. [118] and 1 study [77] used the index by Smith et al. [119]. Overall, DASH was associated with less global cognitive decline in 3 studies (33%) [83,84,88]. Higher DASH score was significantly associated with reduced 4.1-year global cognitive decline (*p* = 0.04) [83], less decline in 3MS scores across 11 years (*p* < 0.001) [88], and reduced likelihood of decline on the MMSE across 19.7 years (*p* = <0.001) [84]. The remaining 6 studies (67%) found no association between DASH and global cognitive decline [9,52,54,57,65,77].

##### MIND Diet and Global Cognitive Decline

The MIND diet was examined by 8 studies in relation to global cognitive decline [9,51,55,65,66,67,77,87]. All used scoring indices from Morris et al. [66] to determine adherence. Three studies (38%) observed positive associations [9,66,77]. Higher adherence to MIND was associated with reduced 4.7-year global cognitive decline in the MAP cohort (β = 0.0106; S.E. 0.002; *p* = <0.0001) [66]. In the SNAC-K cohort (older Swedish adults), higher MIND diet score was associated with less decline on the MMSE over 6 years (β, High vs. low tertile MIND score = 0.126; 95% CI: 0.06, 0.19; *p* = <0.001) [77]. Finally, in Spanish middle-aged adults (SUN cohort) higher MIND score was associated with lower 6-year decline in STICS m score (β per each 1.5 points = 0.27; 95% CI: 0.05, 0.48; *p* = <0.05) [9]. Five studies (62%) [43,51,55,65,67] found no association between MIND adherence and global cognitive decline.

##### AHEI-2010 and Global Cognitive Decline

All 4 studies [9,10,52,73] that investigated AHEI-2010 scores in relation to cognitive decline used the index from Chiuve et al. [120]. One study observed a positive association between AHEI and global cognitive decline over 6 years among the SUN cohort [9]. The remaining 3 studies found no association [10,52,73].

##### Other *a*-Priori DPs and Global Cognitive Decline

Nine studies reported protective effects of other *a*-priori DPs on global cognitive decline including the modified Alternative Healthy Eating index [80], the Healthy Eating Index and the Modified Dutch Healthy Diet 2015 index [69], the Baltic Sea Index [79], a pro-vegetarian diet [9], a plant-based dietary index and a healthy plant-based diet index [90] and a recommended food score [89]. In contrast, there was no association between the Canadian Healthy Eating Index [78], the Australian Dietary Guideline 2013 Index [56] and healthy and traditional Taiwanese DPs [85] and global cognitive decline. In 2 studies greater adherence to *a*-priori unhealthy DPs (a Western diet [85] and an unhealthy plant-based dietary index [90]) were associated with faster cognitive decline over 8–10 years.

##### *A*-Posteriori DPs and Global Cognitive Decline

Fourteen studies investigated adherence to *a*-posteriori DPs (resulting in 29 DPs in total) of which, 8 studies [53,58,59,61,62,71,72,73] reported no association between the derived healthy (termed healthy or prudent) or unhealthy DP (termed western, traditional, high in red meat or butter) and cognitive decline. Unhealthy DP (including an inflammatory DP [70], an iron-related DP [79] and two western DPs [6,68]) were associated with accelerated global cognitive decline among middle-older age adults from UK, China, Sweden and Spain. Healthy DP (including a healthy DP [10], a Mediterranean style DP [68], and prudent DPs [6,77]) were associated with less global cognitive decline among cohorts from UK, Spain and Sweden.

### 3.5. Associations between DPs and Cognitive Disorders

From 83 prospective studies, 28 examined outcomes relating to mild or major cognitive disorders, with follow up ranging from 2.3 to 27 years. A comprehensive overview of these studies is provided in Table S5. In relation to specific outcomes, 7 studies [11,91,92,93,94,95,96] examined risk of MCI, 17 studies [10,45,59,60,87,97,99,100,104,105,107,108,109,110,111,121,122] examined risk of all-cause incident dementia and 7 studies [60,76,92,94,99,102,103,104,105,106,123] investigated risk of AD. Three studies assessed other outcomes, including probable dementia [91], conversion of MCI to AD [92] and vascular dementia [105]. Overall, in relation to dietary exposures, 20 studies (71%) [10,11,45,60,66,76,87,91,92,93,94,96,97,99,104,107,108,109,110,121] examined *a*-priori DPs and 9 studies (32%) examined *a*-posteriori DPs [10,59,95,98,100,102,105,106,111]. Results are summarised below.

#### 3.5.1. DPs and MCI

Seven studies [11,91,92,93,94,95,96] examined DPs (6 studies a-priori DPs [11,91,92,93,94,96]) and 1 study *a*-posteriori DPs [95]) in relation to MCI. Again, the Mediterranean diet was examined most frequently in relation to MCI. In 2 (40%) [92,96] out of 5 studies [91,92,94,96] higher Mediterranean diet score was associated with reduced likelihood of MCI.

In separate studies, greater adherence to DASH [91], MIND [94] and the Chinese Dietary Guidelines Index [93] but not AHEI nor HEI [91], were associated with reduced MCI risk. In the one study [95] which derived 5 *a*-posteriori DPs in a sample of black and white adults aged 45 years and older, there was no notable association between 4 DPs (convenience, sweets/fats, southern-style and plant-based) and MCI, yet higher adherence to an alcohol and salads DP reduced the likelihood of incident MCI (OR, Q5 vs. Q1 = 0.68; 95% CI: 0.56, 0.84; *p* for trend = 0.0005).

#### 3.5.2. DPs and Incident All-Cause Dementia

From 17 studies that investigated the risk of incident all-cause dementia, 12 studies explored *a*-priori DPs [45,60,87,97,99,100,104,107,108,109,110,121] and 6 studies [10,59,98,100,105,111] explored *a*-posteriori DPs. The Mediterranean diet was investigated by 8 studies, including 4 studies [60,104,108,121] which used the MDS [113] and 2 studies [47,109] which used the MedDiet index [114]. In 3 studies (38%) [47,108,109] 2 of which used the MedDiet Index [114], higher Mediterranean diet score was significantly associated with reduced risk of all-cause incident dementia in populations from Spain, Greece and the Netherlands. The remaining 5 studies (62%) that mainly used the population based MDS [113] observed no association of diet score with incident dementia [60,97,104,107,121]. There was also no association between the DASH diet [107,121] or the AHEI [10,107] and dementia. Adherence to the MIND diet reduced the risk of all-cause dementia in a cohort from The Netherlands [109] (HR = 0.79; 95% CI: 0.70, 0.91) and in US-based cohorts (MAP and WHIMS) [87]). Other *a*-priori healthy DPs including the Dutch Dietary Guidelines [109], the HEI [108], a Japanese Diet Index [110] and a Healthy Diet Choice Index [99]) were independently linked with reduced incident dementia.

From the 6 studies deriving *a*-posteriori DPs, 3 reported no association between unhealthy or Western diets and risk of all-cause incident dementia. In contrast, two separate healthy DPs [10,98] were associated with decreased risk of incident dementia with evidence of stronger protective effects among APOE-E4 non-carriers [98]. Furthermore, a Japanese diet [100], and a high soy diet [105], were linked to reduced risk of incident dementia in older Japanese adults.

#### 3.5.3. Adherence to Examined DPs and AD

Risk of AD was examined in relation to *a*-priori DPs in 4 studies [60,76,103,104] and *a*-posteriori DPs in 3 studies [102,105,106]. Two out of 4 studies [76,103] reported a beneficial association between Mediterranean diet and AD risk, whereas the remaining 2 studies [60,104] reported no association. Adherence to the HDI or a low-carbohydrate high protein diet was not associated with incident AD over 12 years in Swedish men [104]. In contrast, the MIND diet but not the DASH diet was associated with reduced likelihood of developing AD in older US adults [103].

Of the 3 studies which investigated *a*-posteriori DPs in relation to AD, each used RRR to derive DPs. A high soy-based diet was not strongly linked with incident AD in an older Japanese population [105]. Older US adults with high adherence to a DP characterised by high intakes of salad dressing, nuts, fish, tomatoes, poultry, fruits, and green leafy and cruciferous vegetables and lower intakes of high-fat dairy products, meat and butter had 38% lower AD risk [HR 0.62; 95% CI 0.43, 0.89] compared to those with low adherence [106]. Furthermore, in a large sample of US women, those with a high sugar diet had 19% higher risk of AD [HR 1.19; 95% CI 1.05, 1.34] than those with a low sugar diet [102].

#### 3.5.4. Adherence to Examined DPs and Other Outcomes Related to Cognitive Disorders

Three studies investigated other outcomes related to cognitive disorders. Higher Mediterranean diet score was related to lower odds of conversion from MCI to AD in older US adults (HR, Q4 vs. Q1 = 0.52; 95% CI: 0.30, 0.91) [92]. A high soy-based diet was protective for vascular dementia among a Japanese cohort (HR, Q4 vs. Q1 = 0.45; 95% CI: 0.22, 0.91) [105]. The third study from Haring et al. [91] examined adherence to several healthy *a*-priori DPs, including Mediterranean diet, DASH diet, AHEI and HEI in relation to outcomes of probable dementia (PD). Higher HEI score, but not the other diet scores, was associated with a reduced likelihood of PD [91].

### 3.6. Overview of Intervention Studies

Ten RCTs explored the effect of DPs on cognitive outcomes as shown in Table S6. The duration of follow-up ranged from 4 weeks to 8.5 years and the sample size ranged from 35-1006 (total *n* = 4477). All participants were reported to be cognitively healthy and mean age ranged from 48–83 years. Four studies [12,13,123,124] were conducted among populations with high cardiovascular risk. Three intervention studies [14,123,124] were based in Australia, 3 from Europe (2 from Spain [12,13], 1 from Sweden [125]), 2 from Asia (1 from Hong Kong [126], 1 from Iran [127]), 1 study from USA [125] and 1 study was conducted across multiple countries [128].

#### 3.6.1. Diet Intervention

Six out of 10 RCTs (60%) examined the effect of a Mediterranean-style diet, [12,13,14,123,124,128]. The other 4 studies examined the MIND-style diet [127], a low-fat diet [125], a healthy DP [126] and an anti-inflammatory DP [129].

#### 3.6.2. Assessment of Outcomes

Repeated neuropsychological test scores were primarily used to determine the change in cognitive performance in most intervention studies [12,13,14,123,124,126,127,128,129]. Only one intervention, nested within a prospective cohort study, examined the effect of diet on cognitive impairment or probable dementia (PD) in US women [125].

#### 3.6.3. Effect of Mediterranean Diet on Cognitive Outcomes

Two out of 6 trials demonstrated improved global or composite cognition in response to Mediterranean diet [12,13]. In a sub-study of the PREDIMED trial (PREDIMED-NAVARRA), older adults with greater adherence to the Mediterranean diet supplemented with nuts or extra virgin olive oil (EVOO) over 6.5 years had less decline in MMSE (Mean Adjusted Difference = 0.62; 95% CI: 0.18, 1.05; *p* = 0.005 and Mean Adjusted Difference = 0.57; 95% CI: 0.11, 1.03; *p* = 0.01), relative to those following a low-fat diet [12]. In a shorter PREDIMED sub-study (4.1 years median follow-up), participants consuming the EVOO-enriched Mediterranean diet had less decline in global cognition (Mean Adjusted Difference = 0.05; 95% CI = −0.11, 0.21), while those eating a nut-enriched Mediterranean diet had improved memory scores (Mean Adjusted Difference = 0.09; 95% CI = −0.05, 0.23) compared to controls. Other trials involving Mediterranean-style diet interventions with shorter durations between 8 weeks [123,124] and 1 year [128], showed less consistent results. Two cross-over studies in Australian adults (aged 45–80 years) demonstrated significant faster processing speed after 8 weeks in response to the Mediterranean diet supplemented with dairy (Med-Dairy) [123] or pork (Med-Pork) [124]. However, the MedLey trial [14] also conducted among Australian adults (aged ≥ 65 years), found no significant effect of a Mediterranean diet on global or individual cognitive domains after 6 months. Finally, the NU-AGE trial which consisted of intervention with an adapted Mediterranean diet (NU-AGE), had beneficial effects on cognition for both intervention and control groups at 1-year follow-up, but reported no significant difference in effects between groups [128].

#### 3.6.4. Effects of Other Diets on Cognitive Outcomes

From the 4 RCTs examining other diets, 3 trials [125,127,129] demonstrated significant effects of healthy DPs on cognitive outcomes. In a trial nested within the Women’s Health Initiative Memory Cohort Study (conducted among US-based women aged 65–79 years) a low-fat diet reduced MCI (HR = 0.65; 95% CI: 0.35, 1.19) and PD (HR = 0.63; 95% CI: 0.19, 2.10) after 8.5 years, compared to controls [125]. A 4-week anti-inflammatory diet (derived from Nordic food guidelines) demonstrated small benefits on cognition in domains of learning, memory and selective attention [129]. Furthermore, in healthy obese women, a 3-month calorie-restricted MIND diet showed positive effects on several tests for verbal memory, working memory, processing speed and attention compared to a standard calorie-restricted diet [127]. In contrast, a healthy diet over 25 months had no impact on cognition among individuals living in old-age hostels in Hong Kong [126].

### 3.7. Risk of Bias in Intervention Studies

Risk of bias assessment using RoB2 is provided in Table S7. Three studies were reported as having some concerns [14,127,128] and 7 studies were reported as having a high risk of bias [12,13,123,124,125,126,129] generally in domains of randomisation, missing outcome data and outcome measurement overall.

## 4. Discussion

### 4.1. Summary of Overall Findings

This review synthesised available prospective and RCT data from the past 16 years up to August 2022 to determine relations between DPs and cognitive outcomes. Findings lend some support to healthy DPs for neuroprotection. However, overall results were mixed across all cognitive outcomes. For example, greater adherence to a healthy DP was associated with better global cognitive performance in 56% of studies, less global cognitive decline in 52% of studies, and reduced risk of incident dementia in 59% of studies, and AD in 57% of studies. The evidence base was also inconsistent for unhealthy DPs with less than half of available studies (*n* = 17) linking unhealthy diet with worse cognitive outcomes.

The DPs explored most frequently in the individual studies were the Mediterranean and DASH diets, but fewer than 50% of studies showed a protective relationship between adherence to these diets and global cognitive decline. Although data for incident dementia was lesser in comparison to that for cognitive decline, *a*-priori DPs, especially the Mediterranean diet appeared to be more strongly protective for AD than for all-cause dementia. As the *a*-priori MIND diet only emerged in 2015,few studies were identified. However, similar to Mediterranean and DASH diets, the results for MIND were also inconsistent with 54% of studies reporting a neuroprotective association. Interestingly, compared to Mediterranean and DASH diets, the MIND diet showed stronger protective effects on risk of incident dementia and AD in mainly older women [87,103] but these findings have not been replicated in other populations. Furthermore, the AHEI did not appear to provide protection against cognitive decline, MCI or dementia risk in most of the available studies. Data from RCTs, while lacking, demonstrated small improvement in cognitive function in response to a Mediterranean style diet in some [12,13] but not all studies [14,128]. Overall, it remains difficult to draw firm conclusions on the efficacy of diet for cognitive health given the limited number of RCTs performed and heterogeneity in findings due to small sample size, variable duration of intervention and differences in both diet intervention and cognitive outcomes measured. Future studies would ideally involve longer term follow-up, to fully investigate the effects of DP adherence on cognitive outcomes across the adult lifespan [18,24]. However, such trials would be expensive to perform and a more pragmatic approach would involve targeting high-risk populations to evaluate the effects of dietary intervention over at least 12 months. An example of such a trial is ongoing to evaluate the effects of the MIND diet on cognition across a 3-year intervention period among a large sample of adults with suboptimal diet (NCT02817074).

Our review findings align somewhat with a previous review [16], suggesting stronger beneficial associations for MIND on cognitive health, followed by the Mediterranean, then DASH diet based on available data. The mechanisms underpinning how diet could benefit cognitive health are not known but likely to involve modulating the oxidative stress and inflammatory pathways implicated in accelerated cognitive decline and AD [130]. Plant-based diets such as MIND and the Mediterranean diet are rich in antioxidants and flavonoids that can reduce systemic inflammatory biomarkers and suppress neuroinflammation [130,131]. However, as we have shown, the evidence base is often inconsistent irrespective of the DP examined. It is possible that the ideal combination of foods for optimal brain health remains elusive and could vary from one population to another. A cultural model reflecting the eating habits of Mediterranean countries may not be appropriate in non-Mediterranean populations. Our review of available data shows that emerging DPs reflecting traditional healthy eating habits of a country, e.g., the Japanese diet [110], and DPs derived from national food guidelines, e.g., Dutch Dietary Guidelines [109] could be important in reducing dementia risk in that target population. Further work to explore culturally appropriate DPs on risk of incident cognitive disorder in different populations is warranted.

Although we attempted to reduce bias in the review by including only prospective data there was substantial between study heterogeneity that precluded a meta-analysis. This was due to methodological inconsistencies in measurement of the dietary exposure (i.e., DP scoring systems used, including cut-off points and adaptations made or dietary intake assessment), cognitive outcomes or robustness of study design and populations assessed, as discussed in more detail below.

#### 4.1.1. Methodological Inconsistencies in Prospective Studies

##### Assessment of Dietary Patterns Exposure

Differences in scoring methods to derive Mediterranean diet score could explain some of the conflict between study findings. The majority (68%) of included studies used the MDS population-based score system [113]. In the MDS, points are allocated based on population sex-specific median intake values per food group, which are then summed to provide an overall score. Beneficially, this provides a clear differentiation between adherers and non-adherers within a population. However, the nature of a population-specific index is problematic as median food intakes will differ between populations. In other words, individuals from different cohorts can have identical scores, yet significantly different eating patterns making it difficult to draw comparisons between studies [132]. In contrast, the MedDiet index [114] is based on absolute food intake values which are then summed to create an overall score, allowing a more meaningful comparison between study populations. Our findings suggest a more consistent pattern of neuroprotection for the MedDiet index, particularly for incident cognitive disorder. Several individual studies also reported making adaptations to *a*-priori DP scoring systems, which made it difficult to draw comparisons between studies. Hence, even when seemingly similar DP score systems used there may be variation in decisions around the types of food components included in overall scores that undoubtedly contributes towards a lack of consistent findings as shown here.

It is also important to note that FFQs were used most frequently to assess dietary intake. While FFQ are useful to estimate long-term dietary intake if undertaken at repeated time-points, and are inexpensive to administer, there are risks of misreporting due to recall bias [133]. Future studies should attempt to counteract such limitations by validating dietary intake measurement through either another qualitative method, or preferably measurement of nutrient biomarkers such as doubly labelled water (energy intake) or urinary nitrogen (protein intake) [133,134,135,136].

##### Assessment of Cognitive Outcomes in the Prospective Studies

Inconsistencies in outcome measurement were related to the use of neuropsychological tests to assess global cognitive function, time points of cognitive assessment and statistical methods used. Studies mainly reported outcomes for global measures of cognition. This may have limited the review results as several studies used clinical screening tests (e.g., MMSE) which have ceiling effects and lack of sensitivity to detect cognitive changes at an early stage [137]. However, there was no substantial difference in the pattern of results between studies using a global screener versus those that used composite measures of global cognition. Several studies [6,7,8,47,60,77,80] showed relations between healthy DPs and better MMSE score suggesting potential benefits of diet for overall cognition.

Furthermore, although most studies reported the number of time points at which neuropsychological tests were employed, this information was difficult to extract from some publications, e.g., for those with variable cognitive measures performed in the sample. As a result, studies often differed in the statistical computation of global cognition, which contributed to the difficulty in drawing comparisons between studies. This highlights the need to standardize methods to assess global cognitive decline, as noted by other reviews [25,138]. This includes standardization of terminology dependent upon time points assessed, (i.e., cognitive performance (2-time points), or cognitive decline (≥3-time points)) to enable better comparison across studies.

##### Population and Duration of Exposure

Few included studies involved participants aged 18–45 years, instead, most were conducted in older adults (>65 years). DPs could have a cumulative effect on cognitive outcomes, as later life is a time point where it is likely the individual has had the longest exposure period to diet. Although DPs are expected to remain relatively stable across time among older adults, most studies (70%) only assessed dietary intake at one-time point, thus diet stability cannot be assumed. Furthermore, given the long latency period for the onset of dementia, there is a high risk of reverse causality in studies conducted among older adults, especially in those with a short follow-up period (<10 years). Therefore, it is possible that dementia pathology may lead to changes in normal eating behaviours and consequently result in spurious associations in prospective studies involving older populations.

Future prospective studies should seek to assess dietary intake regularly across the adult lifespan, this will help to elucidate how long a DP must be adhered to, to promote a healthy cognitive ageing trajectory [30]. More specifically, it will aid understanding when interventions that seek to promote a healthy DP should occur to provide maximum cognitive benefit.

It is also important to note that nearly half (47%) of prospective studies did not provide data on ethnicity or racial status of participants. Given this is a factor which can influence eating habits and predisposition to dementia risk, future studies should consider the impact of ethnic and racial diversity on results [1]. Most evidence in this review was drawn from US cohorts with a paucity of population studies from low and middle-income countries. This reinforces the need for further research into culturally representative DPs to determine commonalities and differences between diets and effects on cognitive decline and dementia risk in diverse populations.

### 4.2. Strengths

This review is strengthened by the inclusion of up to date prospective and RCT data to evaluate the impact of DPs on a range of cognitive outcomes across adulthood. Most prospective studies had follow-up periods ≥5 years and had a relatively large sample size. A broad spectrum of DPs was considered to provide an in-depth understanding about the extent to which adherence to any DP may improve cognition.

### 4.3. Limitations

Review findings should be interpreted with caution due to several limitations. We did not assess associations between specific cognitive domains (e.g., executive function, attention) but rather focused the narrative synthesis on global cognition. Therefore, potential effects of diet on individual cognitive domains were not captured in the current review. Most included studies were prospective, and thus cannot determine causality and are prone to residual confounding. Furthermore, 83% (69/83) of these studies were assessed as low to medium quality. RCTs may be used to draw causal inferences. However, few RCTs were identified, and findings were mixed, with 70% (7/10) of these studies having a high risk of bias, due to issues with randomization, or missing outcome data and outcome measurement. Secondly, we identified substantial heterogeneity between methodologies of included studies, meaning it was not possible to perform a meta-analysis nor determine the extent of publication bias. Thirdly, only studies published in English were considered for inclusion.

## 5. Conclusions

In conclusion, this systematic review found some evidence to link healthy DPs with better cognitive performance, slower cognitive decline, and reduced risk of dementia. However, overall findings were inconsistent. A rationale for such results was provided, with a critical focus on inconsistencies identified. The review discussed the need to standardise measurement and reporting of outcomes related to diet and cognitive function/decline to enable better comparisons between future studies. More studies are needed with adequate follow-up to discern effects of diet on the development of dementia and dementia subtypes. Several gaps were highlighted within the research; (1) a lack of studies which explore culturally appropriate DPs in relation to cognitive decline, MCI and dementia, (2) a lack of adequately powered, well-designed intervention studies, (3) a lack of studies from younger and diverse populations. It is recommended that future studies which seek to explore associations between DPs and cognitive outcomes should conduct follow-up assessments at regular time points including comprehensive measurement of both dietary intake and cognitive outcomes. This would help to better understand the temporal nature of dietary influences on cognition during ageing and the optimal time to intervene with diet interventions to help preserve cognitive functions.

## Data Availability

All data which has been synthesised within this study are presented in the manuscript and Supplementary Materials.

## Supplementary Materials

The following supporting information can be downloaded at: https://www.mdpi.com/article/10.3390/nu15020333/s1; Table S1: Search strategy for systematic review; Table S2: Risk of bias assessment for prospective studies; Table S3: Overview of prospective studies examining associations between change in global cognitive function and DPs; Table S4: Overview of prospective studies examining associations between change in global cognitive decline and DPs; Table S5: Overview of prospective studies examining associations between any mild or major cognitive disorder and DPs; Table S6: Overview of intervention studies examining effects of DPs on cognitive outcomes; Table S7: Risk of bias assessment for intervention studies.
