# Supplementary material for: Whole Dietary Patterns, Cognitive Decline and Cognitive Disorders: A Systematic Review of Prospective and Intervention Studies
Source: Nutrients. 2023 Jan 9;15(2):333. doi: 10.3390/nu15020333 (PMC9865080; doi:10.3390/nu15020333)
Supplement: Supplementary file 1 [file nutrients-15-00333-s001.zip › nutrients-2114989-supplementary.pdf]

Supplementary Table S1. Search strategy for systematic review

NUMBER	SEARCH TERM
1.	“Dietary pattern*”.mp.
2.	“Dietary habit*”.mp.
3.	“Nutrient pattern*”.mp.
4.	Diet therapy/ or caloric restriction/ or diet, carbohydrate loading/ or diet, diabetic/ or diet, carbohydrate-restricted/ or diet, high-protein low-carbohydrate/ or diet, ketogenic/ or diet, fat-restricted/ or diet, gluten-free/ or diet, high-protein/ or diet, mediterranean/ or diet, paleolithic/ or diet, protein-restricted/ or diet, reducing/ or diet, sodium-restricted/ or diet, vegetarian/ or diet, macrobiotic/ or diet, vegan/ or dietary approaches to stop hypertension/
5.	Diet, high-fat/ or diet, western/ or diet, healthy/
6.	“DASH diet*".mp.
7.	“Dietary Approaches to Stop Hypertension*".mp.
8.	“MIND Diet*".mp
9.	"Mediterranean-DASH Intervention for Neurodegenerative Delay*".mp.
10.	"Mediterranean Diet*".mp.
11.	"Low-GI Diet*".mp.
12.	"Low-Fat Diet*".mp.
13.	“Low-Calorie Diet*".mp.
14.	“Healthy Diet*".mp.
15.	“Prudent Diet*".mp
16.	“Healthy Eating Index Diet*".mp.
17.	“Healthy Eating Index*".mp.
18.	“Alternative Healthy Eating Index Diet*".mp.
19.	“Alternative Healthy Eating Index*".mp.
20.	“Inflammatory diet*".mp.
21.	“Diet* Inflammatory Index".mp.

22.	"Food pattern*".mp
23.	“Diet”.ti.
24.	exp Cognition/ or cognition.mp.
25.	exp Cognition Disorders/ or "Cognitive Disorder*".mp.
26.	exp Neuropsychological Tests/ or "Neuropsychological Test*".mp
27.	exp Psychological Tests/
28.	“Psychological Test*".mp.
29.	exp Memory, Long-Term/ or Memory.mp. or exp Memory, Short-Term/ or exp "Memory and Learning Tests"/ or exp Spatial Memory/ or exp Memory Disorders/ or Memory/ or exp Memory, Episodic/
30.	Dementia*.mp. or exp Dementia/ or exp Frontotemporal Dementia/ or exp Dementia, Vascular/ or exp Dementia, Multi-Infarct/ or exp "Mental Status and Dementia Tests”/
31.	“cognitive function*".mp.
32.	“Cognitive impairment*".mp.
33.	“Cognitive Decline*".mp.
34.	“Mild Cognitive Impairment*".mp.
35.	“Cognitive Ageing".mp.
36.	exp Cognitive Aging/
37.	exp Aging/ or "Ageing".mp.
38.	“Age-Associated Cognitive Decline".mp.
39.	“Memory Impairment".mp
40.	“Brain Ag*".mp.
41.	exp Neuroimaging/ or exp Functional Neuroimaging/ or Neuroimaging.mp
42.	“Magnetic Resonance Imaging".mp. or exp Magnetic Resonance Imaging/
43.	“Functional Magnetic Resonance Imaging".mp.
44.	exp Electroencephalography Phase Synchronization/ or exp Electroencephalography/ or Electroencephalography.mp.
45.	Magnetoencephalography.mp. or exp Magnetoencephalography/
46.	“Tomography, X-Ray Computed".mp. or exp Tomography, X-Ray Computed/

47.	“Positron-Emission Tomography".mp. or exp Positron-Emission Tomography/
48.	exp Spectroscopy, Near-Infrared/ or "Functional Near-Infrared Spectroscopy".mp.
49.	“Diffusion Tensor Imaging".mp. or exp Diffusion Tensor Imaging/
50.	“Brain Structure".mp.
51.	“Brain Function*".mp.
52.	“Brain Morphology*".mp.
53.	“Brain Imaging*".mp.
54.	“MRI”.mp.
55.	“PET”.mp.
56.	“CT”.mp.
57.	“MEG”.mp.
58.	“fNIRS”.mp.
59.	“DTI”.mp.
60.	“SPECT”.mp.
61.	“EEG”.mp.
62.	1 or 2 or 3 or 4 or 5 or 6 or 7 or 8 or 9 or 10 or 11 or 12 or 13 or 14 or 15 or 16 or 17 or 18 or 19 or 20 or 21 or 22 or 23
63.	24 or 25 or 26 or 27 or 28 or 29 or 30 or 31 or 32 or 33 or 34 or 35 or 36 or 37 or 38 or 39 or 40 or 41 or 42 or 43 or 44 or 45 or 46 or 47 or 48 or 49 or 50 or 51 or 52 or 53 or 54 or 55 or 56 or 57 or 58 or 59 or 60 or 61
64.	62 and 63
65.	Limit 64 to (english language and humans)
66.	Limit 65 to “all adult (19 plus years)”

Supplementary Table S2. Results of risk of bias assessment for prospective studies using NOS.

Authors, year	Selection				Comparability	Outcome			Overall rating
	Representativeness of the exposed cohort	Selection of the non-exposed cohort	Ascertainment of exposure	Demonstration that outcome of interest was not present at baseline	Comparability of cohorts on the basis of the design or analysis	Assessment of outcome	Was follow-up long enough for outcomes to occur	Adequacy of follow-up of cohorts	
Andreu-Reinon et al., (2021) [108]	★	★	★	★	★★	★	★	★	High
Berendsen et al., (2017) [54]	-	★	-	-	★★	★	★	★	Low
Berendsen et al., (2018) [51]	-	★	-	-	★★	★	★	★	Low
Boumenna et al., (2022) [55]	★	★	★	★	★★	★	★	-	Medium
Cadar et al., (2012) [35]	★	★	-	-	★★	★	★	★	Medium
Charisis et al., (2021) [45]	★	★	★	★	★★	★	★	★	High
Chen et al., (2017) [46]	★	★	-	★	★★	★	★	★	Medium
Chen et al., (2021) [56]	★	★	-	★	★★	★	★	★	Medium
Chen et al., (2022) [57]	★	★	-	★	★★	★	★	★	Medium
Cherbuin & Anstey, (2011) [11]	★	★	-	★	★★	★	★	★	Medium
Chou et al., (2019) [36]	★	★	-	★	★★	★	★	★	Medium
Chuang et al., (2019) [109]	★	★	-	★	★★	★	★	★	Medium
Corley & Deary, (2021) [58]	★	★	-	★	★★	★	★	★	Medium
Daniel et al., (2021) [37]	★	★	-	-	★★	★	★	★	Medium

de Crom et al., (2022) [119]	★	★	-	★	★★	★	★	★	Medium
Dearborn-Tomazos et al., (2019) [59]	★	★	-	★	★★	★	★	★	Medium
Feart et al., (2009) [60]	★	★	★	★	★★	★	★	★	High
Gardener et al., (2015) [61]	★	★	-	★	★★	★	★	★	Medium
Granic et al., (2016) [62]	★	★	★	★	★★	★	★	★	High
Gu et al., (2010) [106]	★	★	★	★	★★	★	★	★	High
Haring et al., (2016) [91]	-	★	-	★	★★	★	★	★	Medium
Hosking et al., (2019) [103]	★	★	-	★	★★	★	★	★	Medium
Hossain et al., (2020) [38]	★	★	★	-	★★	★	★	★	Medium
Hu et al., (2020) [107]	★	★	★	-	★★	★	★	★	Medium
Keenan et al., (2020) [63]	★	★	★	-	★★	★	★	★	Medium
Koyama et al., (2014) [64]	★	★	★	★	★★	★	★	★	High
Larsson et al., (2018) [120]	★	★	-	★	★★	★	★	★	Medium
Liu et al., (2022) [99]	-	★	-	★	★★	★	-	★	Low
Lotan et al., (2022) [65]	-	★	-	★	★★	★	★	★	Medium
Lu et al., (2020) [121]	★	★	-	★	★★	★	★	★	Medium
Lutski et al., (2020) [39]	-	★	★	-	★★	★	★	★	Medium
Mannikko et al., (2015) [40]	★	★	★	★	★★	★	★	★	High
Mattei et al., (2019) [52]	★	★	-	★	★★	★	★	★	Medium
Mazza et al., (2017) [47]	★	★	★	★	★★	★	★	★	High
McEvoy et al., (2019) [41]	★	★	★	-	★★	★	★	★	Medium
Morris et al., (2015) [66]	★	★	-	★	★★	★	★	★	Medium
Morris et al., (2015) [100]	★	★	-	★	★★	★	★	★	Medium
Moustafa et al., (2022) [42]	★	★	-	-	★★	★	★	★	Medium
Mueller et al., (2020) [67]	-	★	-	★	★★	★	★	-	Low

Munoz-Garcia et al., (2020) [9]	★	★	-	★	★★	★	★	★	Medium
Munoz-Garcia et al., (2021) [68]	★	★	-	★	★★	★	★	★	Medium
Nicoli et al., (2021) [94]	★	★	★	-	★★	★	★	★	Medium
Nishi et al., (2021) [48]	★	★	★	★	★★	★	★	★	Medium
Nooyens et al., (2021) [69]	★	★	-	-	★★	★	★	★	Medium
Olsson et al., (2015) [101]	-	★	★	★	★★	★	★	★	Medium
Ozawa et al., (2013) [102]	★	★	-	★	★★	★	★	★	Medium
Ozawa et al., (2017) [70]	★	★	-	★	★★	★	★	★	Medium
Parrott et al., (2013) [71]	★	★	-	★	★★	★	★	★	Medium
Pearson et al., (2016) [104]	★	★	-	★	★★	★	★	★	Medium
Qin et al., (2015) [72]	★	★	★	-	★★	★	★	★	Medium
Richard et al., (2018) [73]	★	★	-	-	★★	★	★	★	Medium
Samieri et al., (2013) [74]	-	★	-	★	★★	★	★	-	Low
Samieri et al., (2013) [75]	-	★	-	★	★★	★	★	★	Medium
Samuelsson et al., (2021) [95]	★	★	★	★	★★	★	★	★	High
Scarmeas et al., (2006) [76]	★	★	★	★	★★	★	★	★	High
Scarmeas et al., (2009) [92]	★	★	★	★	★★	★	★	★	High
Shakersain et al., (2016) [6]	★	★	-	★	★★	★	★	★	Medium
Shakersain et al., (2018) [77]	★	★	-	★	★★	★	★	★	Medium
Shang et al., (2021) [49]	★	★	★	-	★★	★	★	★	Medium
Shatenstein et al., (2012) [78]	★	★	-	★	★★	★	★	★	Medium
Shi et al., (2019) [79]	★	★	★	★	★★	★	★	★	High
Sindi et al., (2018) [96]	★	★	-	-	★★	★	★	-	Low
Smyth et al., (2015) [80]	-	★	-	-	★★	★	★	★	Low
Soldevila-Domenech et al., (2021) [81]	★	★	-	★	★★	★	★	★	Medium

Tanaka et al., (2018) [8]	★	★	-	★	★★	★	★	★	Medium
Tangney et al., (2011) [82]	★	★	-	★	★★	★	★	★	Medium
Tangney et al., (2014) [83]	★	★	-	★	★★	★	★	★	Medium
Tomata et al., (2016) [97]	★	★	-	★	★★	★	★	★	Medium
Tong et al., (2021) [84]	★	★	★	-	★★	★	★	★	Medium
Trichopoulou et al., (2015) [7]	★	★	★	★	★★	★	★	★	High
Tsai, (2014) [85]	★	★	-	★	★★	★	★	-	Medium
Tsivgoulis et al., (2013) [105]	★	★	-	★	★★	★	★	★	Medium
van lent et al., (2021) [43]	★	★	-	★	★★	★	★	★	High
Vercambre et al., (2012) [86]	-	★	-	-	★★	★	★	★	Low
Vu et al., (2022) [87]	★	★	-	★	★★	★	★	★	Medium
Wade, Elias & Murphy, (2019) [44]	★	★	-	★	★★	★	★	★	Medium
Walters et al., (2018) [50]	★	★	-	★	★★	★	★	★	Medium
Wengreen et al., (2009) [89]	★	★	-	★	★★	★	★	★	Medium
Wengreen et al., (2013) [88]	★	★	-	★	★★	★	★	★	Medium
Zhang et al., (2021) [93]	★	★	★	-	★★	★	★	-	Medium
Zhu et al., (2022) [90]	★	★	-	★	★★	★	★	★	Medium

Supplementary Table S3. Overview of prospective studies examining associations between DP adherence and change in cognitive function

Authors, year, *study name*, country	Study characteristics				Diet measures		Cognitive measures		Results		
	Follow-up time (years)	N =	Population characteristics	Mean age at baseline, y	Food intake assessment; time point(s) assessed	DP(s) examined, (scoring reference or a-posteriori)	Cognitive tests	Timepoint(s) of cognitive assessment	Primary outcome(s) of interest	Main findings from primary outcome of interest	Covariates
Cadar et al., (2012) [35], UK.	17+	1,018	Individuals aged 46-64 (48.1% male).	43	5-d Diet Diary; 2 time point(s).	1. Healthy food choice score, Cadar et al., (2012).	15-item word learning task; Letter search task.	2	Verbal memory score	Significant association between adherence to healthy food choice score and reduced rate of change in verbal memory score across 17+ years (β, = 0.70; 95% CI: 0.02,1.38; P = 0.043).	Sex, father social class, childhood cognitive ability, education, midlife household occupational social class, depression, change in other lifestyle behaviours.
Chen et al., (2017) [46], Taiwan.	2	475	Individuals aged 65 and older (28.5% female).	73	44-item FFQ; 1 time point.	1. Vegetable, (A-posteriori via EFA).<br>2. Meat, (A-posteriori via EFA).<br>3. Traditional, (A-posteriori via EFA).	MoCA-Taiwan Version; Wechsler Memory Scale–Third Edition; Digit-span reverse; Verbal fluency tests; Trail-making tests A and B.	2	Global cognition (computed via MoCA-Taiwan version).	No significant association between adherence to vegetable DP, meat DP or traditional DP and change in global cognition across 2 years. However, significant associations were found between DPs and decline in specific-domains.	Age, sex, education, energy intake, physical activity, depression, APOE e4 status, supplement use, disease history (diabetes, HTN, hyperlipidaemia)

Cherbuin & Anstey, (2011) [11] *PATH Study,* Australia.	4	1,528	Community dwelling, middle-aged adults (51.2% female).	60-64	183-item FFQ; 1 time point.	1.Mediterranean diet, from Trichopoulou et al. (2003).	Immediate recall; Delayed recall; Digits backward; Spot-the-word; Symbol-digit modalities test; Simple reaction time; Complex reaction time	2	Global cognition (computed via sum of scores from all tests).	No significant association between adherence to Mediterranean diet and change in global cognition across 4 years among normal individuals.	Age, sex, education, APOE e4, BMI, physical activity, stroke, diabetes, hypertension, total caloric intake.
Chou et al., (2019) 36]; *Taiwan Initiative for Geriatric Epidemiological Research;* Taiwan.	2	436	Individuals aged 65 and older, living in the community (53% female).	72.5 ± 5.2	44-item FFQ; 1 time point	1. Modified Alternative Healthy Eating Index (mAHEI) from Dehghan et al. (2012)	MoCA-Taiwan Version (MoCA-T); Wechsler Memory Scale–Third Edition; Digit-span forward and backward; Verbal fluency tests; Trail-making tests A and B.	2	Global cognition (computed via MoCA-Taiwan version)	Significant association between adherence to mAHEI and reduced odds of decline in MoCA-T across 2 years (OR, T_3 vs T_1 = 0.54; 95% CI: 0.31, 0.95; P = 0.03).	Age, sex, education, apoe e4, baseline cognition, total caloric intake and depressive symptoms.
Lutski et al., (2020) [39]; *BIP Study;* Israel.	4.8 ± 1.3	200	200 men recruited with history of CVD from medical centres (100% male).	57.3 ± 6.3	4-day food diary; 1 time point.	1. Mediterranean diet, adapted from Trichopoulou et al. (2003).	Memory tests; Executive function tests; Visuo-spatial processing tests and attention tests.	2	Global cognition (computed via sum of scores from all tests).	Significant association between adherence to the Mediterranean diet and change in global cognition across 4.8 years (β, T_3 vs T_1 = -0.23; 95% CI: -0.43, -0.04; P = 0.021).	Age, education, birth place, caloric intake, height, smoking, BMI, physical activity, NYHA class, history of hypertension, past myocardial infarction, CRP, insulin resistance, diastolic blood pressure.

Mannikko et al., (2015) [40]; Finland.	4	1,140	Healthy older adult (55-74) men and women residing in Kuopio in 2002 (50.3% female).	66.1 ± 5.2	4-day food diary; 2 time points.	1. Nordic diet, Adapted from Kanerva et al. (2013).	Standardised version of CERAD (includes verbal fluency, modified Boston naming test, Word list learning, constructional praxis, word list recall and word list recognition discriminability); MMSE.	2	CERAD-TS; MMSE.	No significant association between the Nordic diet and change in standardised CERAD scores or MMSE scores across 4 years.	Age, sex, education, study group, total caloric intake, baseline CERAD total score or MMSE, depression, smoking, Vo2 Max, antihypertensive medication, lipid-lowering medication and antidiabetic medication.
Mazza et al., (2017) [47]; Italy.	1	214	Community-dwelling, white individuals aged ≥65 years old.	70 ± 4	24-hr dietary recall and 7-day diet record; 1 time point.	1. Legumes, (A-posteriori via PCA)<br>2. Plant proteins, (A-posteriori via PCA).	Validated Italian version of MMSE and ADAS-cog.	2	MMSE; ADAS-Cog.	Significant association between adherence to the legumes DP and MMSE (β = 0.25; 95% CI: 0.07, 0.44; P = 0.007) and ADAS-Cog (β = -0.10; 95% CI: -0.79, -0.30; P = 0.006) after 1 year of follow up. Significant association between plant proteins DP and ADAS-cog after 1 year of follow up (β = 0.584; 95% CI: 0.04, 0.42; P = 0.04).	
McEvoy et al., (2019) [41]; *CARDIA;* USA.	5	2,621	Randomly selected black and white healthy adults aged 18-30 at baseline in 1985-86 (57% female).	25.2 ± 3.5	Diet intake history assessed by trained interviewer; 3 time points.	1. Mediterranean diet, from Panagiotakos et al. (2007).<br>2. DASH, from Folsom, Parker,	Rey auditory verbal learning test; Digit symbol substitution test; Stroop test; MoCA.	2	Cognitive function (computed via sum of scores from all tests).	Significant association between adherence to the Mediterranean diet and the APDQS and change in	Sex, age, race (white, black), education (years), smoking, body mass index, diabetes mellitus, physical activity, and total energy intake.

						and Harnack (2007).<br>3. A Priori Diet Quality Score (APDQS), Sijtsma et al. (2012).				cognitive function across 5 years when compared by dietary tertile. No significant association between adherence to the DASH diet and change in cognitive function across 5 years when compared by dietary tertile.	
Trichopoulou et al., (2015) [7] *EPIC-Greece,* Greece.	6.6 Range 5.1-8.2 y	401	Participants were healthy individuals residing within Attica aged ≥60 at baseline (64% female).	66	Interviewer administered 150-item validated FFQ; 1 time point.	1. Mediterranean diet, from Trichopoulou et al. (2003).	MMSE: Mild cognitive decline defined as change in MMSE (−4 to −1) vs ≥0. Substantial cognitive decline defined as change in MMSE ≤ −5 vs ≥0.	2	Mild cognitive decline (defined as change in MMSE (−4 to −1, vs ≥0)); Substantial cognitive decline (defined as change in MMSE ≤−5 vs ≥0).	Significant association between adherence to the Mediterranean diet and risk of mild cognitive decline (OR, T_3 vs T_1 = 0.46; 95% CI: 0.25, 0.87; P = 0.0012) and risk of substantial cognitive decline (OR, T_3 vs T_1 = 0.34; 95% CI: 0.13, 0.89; P = 0.025).	Sex, age, years of education, BMI, physical activity, smoking status, diabetes, hypertension, cohabiting, and total caloric intake.
Walters et al., (2018), [50] USA.	3	70	Cognitively and clinically normal middle-aged adults (69% female).	49 ± 8	Harvard Willett Semi-quantitative FFQ; 1 time point.	1. Mediterranean diet, from Walters et al., (2018).	Immediate and delayed recall of a paragraph and immediate and delayed recall of paired associates; Wechsler Adult Intelligence Scale (WAIS) digit	2	Global cognition (computed via sum of scores from all tests).	No association between adherence to the Mediterranean diet and change in global cognition across 2 years.	Age, sex, education, diet, exercise and intellectual activity scores, BMI, QUICKI scores, APOE E4, hypertension.

							symbol substitution and WAIS vocabulary.				
Wade, Elias & Murphy, (2019) [44] Maine-Syracuse Longitudinal Study, USA.	5	530	Community-dwelling adults (62.8% female).	61.6 ± 11.8	Nutrition and Health Questionnaire (NHQ) similar to FFQ; 1 time point.	1. Mediterranean diet from Sofi et al., (2013) .	Origin Wechsler Memory Scale-Revised; Hopkins verbal learning test; Visual reproductions-immediate recall; Visual reproductions-delayed recall; Origin Wechsler Adult Intelligence Scale III; Origin Wechsler Adult Intelligence Scale; Hooper visual organisation; Trail making A; Trail making B; Controlled oral word associations.	2	Global cognition (computed via sum of scores from all tests).	Among individuals ≥70 years (N = 139), significant association between adherence to Mediterranean diet and change in global cognitive function across 5 years (β = 0.71, 95% CI: 0.07, 0.45; P = 0.01). However found no association among individuals aged <70 years.	Gender, cognitive score at wave 6, age, education, ethnicity, systolic blood pressure, total cholesterol, total serves of food, total cigarettes, total MET hours.
Charisis et al., (2022) [45], *Hellenic LongitudinaI Investigation of Aging and Diet (HELIAD)*, Greece.	3.1 ± 0.9	1,046	Community dwelling individuals aged over 64 (40.3% male).	73.1 ± 5	Semi-quantitative, validated FFQ; 1 time point.	1. Mediterranean diet, Panagiotakos et al., (2007) [1]	MMSE; medical college of Georgia complex figure test; Greek verbal learning test; Greek version of the Boston naming test-short form; complex ideational material subtest; judgement of line orientation abbreviated form; clock drawing test; trail making test; trail making test part B; Greek	2	Global cognition (computed via sum of scores from all tests).	1. Significant positive association between Mediterranean diet and change in global cognition across follow-up (β = 0.003; 95% CI = 0.001, 0.006; P = 0.007).	Age, sex, education, energy intake, BMI, clinical comorbidities, MCI at baseline, evaluation centre, physical activity, ApoE genotype.

							multiple choice vocabulary test.				
Daniel et al., (2021) [37], *Multi-Ethnic Study of Atherosclerosis (MESA)*, USA.	Range = 9	4,169	Adults recruited to MESA from across 6 US communities (53% female).	60.4 ± 9.5	120-item block style FFQ; 1 time point.	1. DASH, Fung et al., (2008) [4].	Cognitive abilities screening instrument (CASI); digit symbol coding and digit span.	2	Global cognition (sum of CASI score)	1. No association between adherence to DASH DP and change in global cognition (β = − 0.16; S.E = 0.16; P = 0.37).	Age, sex, race/ethnicity, education, income, APOE E4, BMI, smoking, alcohol intake, depressive symptoms, physical activity, energy intake, diabetes, diabetes medications, systolic and diastolic blood pressure, anti-hypertensive medications, stroke, HDL, LDL, total cholesterol.
Hossain et al., (2020) [38], *Healthy Aging in Neighborhoods of Diversity across the Life Span (HANDLS)*, USA.	4.7	225	Adults from HANDLS, with genetic data and aged over 50 years old (55% female).	Men = 57.1 ± 0.5; Women = 56 ± 0.8,	24 hr dietary recalls; 2 time points.	1. Diet quality score, authors used HEI-2010 from Guenther et al., (2005) and DASH DP from Mellen et al., (2008) combined via PCA analyses.	MMSE; CVLT List A; CVLT-Free Recall Long Delay; Benton Visual Retention Test (BVRT); Brief Test of Attention; Trailmaking Test A & B; Digits Span Forward & Backward; Clock Command Test; Identical Pictures Test; Card; Rotation Test; Animal Fluency Test.	2	MMSE (selected for purposes of review)	1. No significant association between diet quality DP and change in MMSE across follow-up (β = -0.01; S.E = 0.04).	Age, sex, race, education, poverty status, BMI, drug use, smokeing status, T2DM, hypertension, dyslipidaemia, CVD, non-fatal myocardial infarction or atrial fibrillation, inflammatory disease and use of NSAIDS.
Moustafa et l., (2022) [42], *HCHS/SOL & SOL-INCA*, USA.	7	6,321	Hispanic and Latino adults living in the US from SOL-INCA and HCHS/SOL (57.8% female).	56.1 ± 0.18	24-hour dietary recall; 2 time points.	1. Mediterranean DP, Mattei et al., (2017) and Ye et al., (2013).	Brief Spanish English Verbal Learning Test; B-SEVLT Recall; Word fluency; Digit Symbol substitution Test (DSST).	2	Global cognition (sum of tests).	1. No association between either Mediterranean DP and change in global cognition across 7 years for either low adherence group (P for trend = 0.32).	Age, sex, education, language preference, hypertension, stroke or transient ischaemic attack history, smoking status, health insurance, household income, US-born, physical activity, kidney function, BMI and number of vascular risk factors.
Nishi et al., (2021) [48],	2	6,647	Recruited participants were aged 55-75 years	65.0 ± 4.9	143-item semi-quantitative validated	1. Mediterranean	MMSE; Clock drawing test; VFT-a and VFT-p; Trail	2	Global cognition (computed	1. No association between adherence to the	Age, sex, intervention group, centre size, cognitive test score at baseline, education, civil status, smoking habits,

PREDIMED+, Spain.			with overweight or obesity, meeting ≥3 criteria for metabolic syndrome (48.4% female).		FFQ; 1 time point.	DP, Schröder et al., (2011) [6].<br><br>2. DASH, Fung et al., (2008) [4].<br><br>3. MIND, Morris et al., (2015) [2].	making tests A & B; Forward and backward recall digit span tests of Weschler Adult Intelligence Scale-III.		via sum of all test scores).	Mediterranean DP and 2 year change in global cognition (β, T_3 vs T_1 =0.033; 95% CI = -0.009, 0.075; P for trend = 0.095). However Mediterranean DP was associated with MMSE (β, T_3 vs T_1 = 0.068; 95% CI = 0.017, 0.125; P for trend = 0.014).<br><br>2. No association between adherence to the DASH DP and 2 year change in global cognition (β, T_3 vs T_1 = -0.027; 95% CI = -0.067, 0.014; P for trend = 0.154).<br><br>3. No association between adherence to the MIND DP and 2 year change in global cognition (β, T_3 vs T_1 = 0.023; 95% CI = -0.017, 0.063; P for trend = 0.367).	hypertension, hypercholesterolaemia, depressive symptomology, [physical activity at baseline, total energy intake.
Shang et al., (2021) [49], *China Health and Nutrition Survey*, China.	7 (median)	2,307	Individuals living within selected areas of China aged 55 and older	63.3 ± 7.0	3-day food diary; Multiple time points.	1. Beans-mushroom (a-posteriori via EFA) 2. Dairy-fruits-fast foods	TICS-m; Immediate recall of a 10-word list and delayed recall of a 10-word list; Counting	2	Global cognition (computed via sum of	1. Positive association between beans-mushroom DP and change in	Education, living area, follow-up years, smoking, alcohol, physical activity, global cognitive score, diabetes, BMI, blood pressure, intake of energy,

			at follow-ups (50.8% female).			(a-posteriori via EFA) 3. Grains-vegetables-pork (a-posteriori via EFA) 4. Plant-based foods (a-posteriori via EFA) 5. Beverages-nuts (a-posteriori via EFA)	backward from 20; Serial 7 subtraction.		scores from all tests).	global cognition (β, Q_5 vs Q_1 = 0.17; 95% CI = 0.05, 0.30; P = 0.009).<br><br>2. No association between dairy-fruits-fast foods DP and change in global cognition (β, Q_5 vs Q_1 = 0.03; 95% CI = -0.10, 0.16; P = 0.0609).<br><br>3. No association between grains-vegetables-pork DP and change in global cognition (β, Q_5 vs Q_1 = -0.04; 95% CI = -0.18, 0.10; P = 0.74).<br><br>3. No association between plant-based foods DP and change in global cognition (β, Q_5 vs Q_1 = -0.02; 95% CI = -0.10, 0.14; P = 0.50).<br><br>3. Positive association between beverages-nuts DP and change in global cognition (β, Q_5 vs Q_1 = 0.18; 95%	sodium, potassium, carbohydrates, fat, protein and fibre.

										CI = 0.06, 0.29; P = <0.0001).	
Van Lent et al., (2021) [43], *Framingham Heart Study Offspring*, USA.	6.6 ± 1.1	1,584	Offspring of original Framingham Birth Cohort (45.7% male).	61 ± 9	126-item semi-quantitative FFQ; 2-3 time points.	1. MIND DP, Morris et al., (2015) [2].	Visual Reproductions Delayed recall; Logical Memory Delayed Recall; Wechsler Memory Scale; Similarities from Wechsler Adult Intelligence Scale; Trail Making Test A & B; Hooper Visual Organisation Test.	2 time points.	Global cognition (computed via sum of scores from tests).	1. No association between MIND adherence and change in global cognition across 6.6 years follow-up (β = -0.002 ± 0.02; P = 0.87). However MIND adherence was associated cross-sectionally with better global cognitive function at baseline (β = 0.03 ± 0.01; P = 0.004).	Age at neuropsychological exam, age squared at exam, APOE E4 status, calorie intake, education, time from exam to outcome, BMI, physical activity index, smoking, diabetes, prevalent cardiovascular disease, depressive symptoms, anti-hypertensive medication usage, systolic blood pressure and total to HDL cholesterol ratio.

Supplementary Table S4. Overview of prospective studies examining associations between DP adherence and cognitive decline

Authors, year, *study name*, country	Study characteristics				Diet measures		Cognitive measures		Results		
	Follow-up time (years)	N =	Population characteristics	Mean age at baseline, y	Food intake assessment; time point(s) assessed	DP(s) examined, (scoring reference or a-posteriori)	Cognitive tests	Timepoint(s) of cognitive assessment	Primary outcome(s) of interest	Main findings from primary outcome of interest	Covariates
Akbaraly et al., (2019) [10]; *Whitehall II;* UK.	24.8	8,225	Civil servants aged 35-55 at baseline (30.9% female).	50.2 ± 6.1	127-item FFQ; 3 time point(s).	1. AHEI, from Chiuve et al. (2012).<br>2. Healthy DP, (a-posteriori via PCA).<br>3. Western DP, (a-posteriori via PCA).	20-word free recall test; Alice Heim 4-1 test; phonemic fluency tests; semantic fluency tests.	≥1-6	Global cognition (computed via sum of scores from all tests).	Significant association between adherence to a healthy DP and change in global cognition across 18 years (β = -0.03; 95% CI: -0.05, -0.01; P = 0.007). No associations found between adherence to AHEI or western food pattern.	Age, sex, race/ethnicity, total caloric intake, education level, occupational position, marital status, smoking status, physical activity level, alcohol consumption (apart from in AHEI score is the exposure because alcohol is one of the components of AHEI score), hypertension, type 2 diabetes, body mass index, dyslipidemia, depressive symptoms, coronary heart disease or stroke, use of any cardiovascular disease medication, and covariate interactions with time.
Berendsen et al., (2017) [54]; *Nurses' Health Study;* USA.	4.1	16,144	Women, ≥70 years old and free of stroke (100% female).	74.3 ± 2.3	116-item FFQ; 5 time point(s).	1. DASH, from Fung et al. (2008).	Immediate recalls of EBMT; Delayed recalls of EBMT; Immediate recalls of TICS-10 word list; Delayed recalls of TICS-10 word list; Category fluency test; Digit-span backward test.	≥1-4	Global cognition (computed via sum of scores from all tests); TICS score.	No significant association between adherence to DASH and change in global cognitive score or TICS score across 4.1 years.	Age, education, energy intake, physical activity, BMI, tobacco use, alcohol intake, depression, multivitamin-use, diabetes, hypertension, hypercholesterolemia, myocardial infarction.

Berendsen et al., (2018) [51] *Nurses' Health Study*, USA.	6	16,058	Women, ≥70 years old and free of stroke (100% female).	74.3 ± 2.3	116-item FFQ; 5 time point(s).	1. MIND, from Morris et al. (2015).	Immediate recalls of EBMT; Delayed recalls of EBMT; immediate recalls of TICS-10 word list; Delayed recalls of TICS-10 word list; Category fluency test; Digit-span backward test.	≥1-4	Global cognition (computed via sum of scores from all tests); TICS score.	No significant association between adherence to MIND DP and change in global cognition and TICS score across 6 years.	Age, education, physical activity, caloric intake, alcohol intake, smoking status, multivitamin use, BMI, depression and history of high blood pressure, hypercholesterolemia, myocardial infarction, and diabetes mellitus.
Morris et al., (2015) [66]; *Rush Memory and Aging Project (MAP)*; USA.	4.7	960	Participants from retirement communities and senior public housing (75% female).	81.4 ± 7.2	144-item FFQ;	1. MIND from Morris et al. (2015).	Immediate and delayed memory test from Story A logical memory subset of the wechsler memory scale-revised; Immediate and delayed word recall of the CERAD word list; CERAD word list recognition; Immediate memory test of East Boston Story; Delayed memory test of East Boston Story; Verbal fluency from CERAD; 15-item version of Boston Naming Test; 15-item reading test; 15-item Judgement of Line orientation; 16-item Standard progressive matrice; Oral version of the symbol digit modalities test; Number	≥2-10	Global cognition (computed via sum of scores from all tests).	Significant association between adherence to the MIND diet and rate of change in global cognitive score across 4.7 years (β = 0.0106; S.E. 0.003; P = <0.0001).	Age, Mind diet score, sex, education, participation in cognitive activities, APOE-ε4 (any ε4 allele), smoking history (current, past, never), physical activity hours per week, total energy intake, history of stroke, myocardial infarction, diabetes, hypertension and time and interaction terms between each covariate and time.

							comparison; 1 indices from Stroop Neuropsychological Screening Test; Digit Span sub tests forward and backward of the Weschler Memory Scale-Revised; Digit Span subtests- backward of the Weschler Memory Scale-Revised; Digit Ordering.				
Feart et al., (2009) [60]; *Three City Study;* France.	5	1,410	Community-dwelling individuals aged 65 or older from electoral rolls (57.8-60.1% female)	75.9	40-item FFQ and 24-hour diet recall; 1 time point.	1. Mediterranean diet from Trichopoulou et al. (2003).	MMSE; Benton visual retention test (BVRT); Free and cued selective reminding test (FCSRT); Isaacs set test (IST).	≥2-5	MMSE; BVRT; FCSRT; IST.	Significant association between adherence to the Mediterranean diet and fewer errors on MMSE across 5 years (Per each 1 point increase in the Mediterranean diet score: β = -0.006; 95% CI: -0.01, -0.0003; P = 0.04), but not with change in performance on BVRT, FCSRT or IST across 5 years.	Age, sex, education, marital status, total energy intake, practice of physical exercise, taking 5 medications/d or more, Centre for Epidemiological Studies-Depression scale score, and ApoE e4 and their interaction with time, body mass index, hypertension, hypercholesterolemia, diabetes, and tobacco use and their interaction with time.
Dearborn-Tomazos et al., (2019) [59]; *Atherosclerosis Risk in Communities Study (ARIC);* USA.	20	13,588	Community-dwelling individuals aged 45-64 years old and living in selected communities (55.8% female).	54.6 ± 5.7	66-item FFQ; 1 time point	1. Western, (A-posteriori via PCA).<br>2. Prudent, (A-posteriori via PCA).	Digit Symbol Substitution Test; Word Fluency Test; Delayed Word Recall Test.	3	Change in cognitive z score (computed via sum of scores from all tests).	No significant association between adherence to the western DP or prudent DP and change in	Age, sex, education, race–field centre, and total energy intake for model 1 and APOE ε4 status, alcohol use history, smoking history, activity level, body mass index, total cholesterol, prevalent coronary heart disease,

										cognitive z score across 20 years.	history of hypertension, diabetes, and stroke.
Gardener et al., (2015) [61]; *Australian Imaging, Biomarkers and Lifestyle study of ageing (AIBL)*; Australia.	3	527	Healthy, cognitively normal individuals aged 60 or above (39.8% male)	69.3 ± 6.4	74-item FFQ; 1 time point	1. AusMedi, Adapted from Trichopoulou et al. (2003).<br>2. Western, (A-posteriori via PCA).<br>2. Prudent, (A-posteriori via PCA).	Logical memory II; California verbal Learning test-II delayed recall; California verbal learning test-ii d'; Rey complex figure test 3 minute delay; Rey complex figure test 30 minute delay; Rey complex figure test recognition; Stroop C/D; Controlled oral word association task; Fruit and furniture switching test; Category fluency test; Boston naming test; Digit span forwards and backwards; Stroop dots time; Digit symbol coding; Rey Complex Figure Test; Clock test.	3	Global cognition (computed via sum of scores from all tests).	No significant association between adherence to the AusMedi, Western or Prudent DPs and change in global cognition over 3 years.	Age, baseline diet score, time, APOE genotype, BMI, country of birth, (Australia or other), years of education (⩽12 years or 412 years), past smoking status, energy intake, history of angina, stroke, hypertension, heart attack and diabetes.
Mattei et al., (2019) [52]; *Boston Puerto Rican Health Study*; USA	2	913	Middle-aged and older Puerto Rican adults living in mainland USA (Boston) (69.5-73.6% female).	45-75	Validated FFQ; 1 time point.	1. Mediterranean diet, Adapted from Trichopoulou et al. (2003).<br>2. Healthy eating index 2005 (HEI-2005) from Guenther,	MMSE; 16-word list learning test; Stroop test; Digit span forward and backward test; Verbal fluency; Clock drawing; Figure copying.	3	Global cognition (computed via sum of scores from all tests).	All subjects were analysed by T2DM status. Significant association between adherence to the Mediterranean diet and change in global cognition across 2 years	Age, education, birth place, caloric intake, height, smoking, BMI, physical activity, NYHA class, history of hypertension, past myocardial infarction, CRP, insulin resistance, diastolic blood pressure.

						Reedy, and Krebs-Smith (2008).<br><br>3. Alternative healthy eating index 2010 (AHEI-2010), from Chiuve et al. (2012).<br><br>4. DASH, from Fung et al. (2008).				among individuals with T2DM (β = 0.027; S.E. = 0.011; P = 0.016). No significant association between adherence to HEI, AHEI or DASH and change in global cognition across 2 years among individuals with T2DM. However, no significant association between adherence to the Mediterranean diet, the HEI, AHEI or DASH and change in global cognition across 2 years among individuals without T2DM.	
Munoz-Garcia et al., (2019) [9]; *Seguimiento Universidad de Navarra (SUN)*; Spain.	6 ± 3	806	Participants were university graduates from Navarra University (68.8% male).	61 ± 6	136-item semi-quantitative FFQ; 1 time point.	1. Mediterranean diet, Trichopoulou et al. (2003).<br><br>2. MEDAS, from Schröder et al. (2011).<br><br>3. DASH, from Fung et al. (2008).	Spanish version of the modified Telephone Interview for Cognitive Status (STICS-m).	3	STICS-m	No significant association found between adherence to the Mediterranean diet, the MEDAS, DASH or Pro-vegetarian diet and change in STICS-m score across 6 years. Significant association between	Age, sex, follow-up time until baselines STICS-m, years of university education, APOE E4, smoking and number of package-years, total caloric intake, physical activity, BMI, alcohol intake, depression, hypertension, high cholesterol, low HDL cholesterol, cardiovascular disease, T2DM, atrial fibrillation.

						4. MIND, from Morris et al. (2015).<br><br>5. AHEI-2010, from Chiuve et al. (2012).<br><br>6. Pro-vegetarian diet (PVD) from Martínez-González et al. (2014).				adherence to AHEI-2010 and MIND and change in STICS-m score across 6 years (β, per each 9 points = 0.25; 95% CI: 0.04; 0.45; <0.05 and β per each 1.5 points = 0.27; 95% CI: 0.05, 0.48; P = <0.05, respectively).	
Ozawa et al., (2017) [70]; *Whitehall Study;* UK.	10	5,083	Civil servants aged 35-55 at baseline (28.7% male).	56	127 item FFQ; 2 time points.	1. Inflammatory DP, (A-posteriori via RRR).	Alice Heim 4-I; Short-term verbal memory; Verbal fluency; MMSE (only used in 2002-2004 and 2007-2009).	3	Global cognition (computed via change in sum of scores from all tests except MMSE); MMSE.	Significant association between adherence to inflammatory DP and greater change in global cognition across 10 years when comparing those in T3 (β = -0.35; 95% CI: -0.38, -0.32) to those in T1 (β = -0.31 SD; 95% CI: -0.33, -0.28) (P = 0.04).<br><br>However, no significant association between inflammatory DP and decline of ≥3 points in MMSE score when comparing T3 to T1.	Age, sex, ethnicity, education and total energy intake, BMI,diabetes, HTN, smoking, leisure time physical activity, ethnicity, occupational position, education, smoking history.

Qin et al., (2015) [72]; *China Health and Nutrition Survey*; China.	5.3	1,650	Older community-dwelling individuals residing within China (50% female).	63.5	24-hr diet recall over 3 days by trained interviewers;1 time point.	1. Mediterranean diet, Adapted from Trichopoulou et al. (2003).<br><br>2. Wheat-based Diverse Diet, (A-posteriori via PCA).<br><br>3. Rice/pork pattern, (A-posteriori via PCA).	Modified Telephone Interview for Cognitive Status (TICS)	3	Composite cognitive score; Global cognition.	No association between adherence to the Mediterranean diet, wheat-based DP or rice-pork DP and change in composite cognitive scores or global cognition across 5 years. When stratified by age, significant associations found between greater adherence to Mediterranean diet and reduced difference in rate of change in both composite cognitive score and global cognitive score among individuals aged ≥65 years old.	Age, sex, region, urbanisation index, education, annual household income per capita, physical activity, current smoking, total caloric intake, time and interaction between time and all covariates, BMI, hypertension.
Samieri et al., (2013) [74] *Women's Health Study,* USA.	4	6,174	Participants were subset of participants from the Women's Health Study aged ≥65 y (100% female).	72	131-item semi-quantitative FFQ; 1 time point.	1. Mediterranean diet, adapted from Trichopoulou et al. (2003).	Telephone Interview for Cognitive Status (TICS); Immediate and delayed recalls of East Boston Memory Tests (EBMT); Delayed recall of the TICS 10-word list; Category fluency test; Digit span backward.	3	Global cognition (computed via sum of scores from all tests).	No significant association between adherence to the modified Mediterranean diet scores and change in global cognition across 4 years.	Treatment arm, age at initial cognitive testing, Caucasian race, high education, high income, energy intake, physical activity, BMI, smoking, diabetes, hypertension, hypercholesterolemia, hormone use, and depression.
Samieri et al., (2013) [75]	6	16,058	Participants were subset of	74.3	116-item semi-quantitative	1. Mediterranean	Telephone Interview for	3	Global cognition	No significant association	Age, education, total caloric long-term intake, physical activity, BMI,

Women's Health Study, USA.			participants from the Women's Health Study aged ≥65 y (100% female).		FFQ; 5 time points.	diet, adapted from Trichopoulou et al. (2003).	Cognitive Status (TICS); Immediate and delayed recalls of East Boston Memory Tests (EBMT); Delayed recall of the TICS 10-word list; Category fluency test; Digit span backward.		(computed via sum of scores from all tests).	between adherence to the modified Mediterranean diet and change in global cognition across 6 years.	smoking, depression, multivitamin use, diabetes, HN, hypercholesterolemia, myocardial infarction.
Scarmeas et al., (2006) [76]; *WHICAP*; USA.	4 ± 3	2,258	Community-dwelling individuals aged 65 or older without dementia residing in Northern Manhattan (32% male).	77.2 ± 6.6	Interviewer administered 61-item validated semi-quantitative FFQ; 1 time point.	1. Mediterranean diet, from Trichopoulou et al. (2003).	Selective Reminding Test; Benton Visual Retention Test; 10 items from the MMSE; Rosen Drawing Test; Benton Visual Retention Test Matching; Boston Diagnostic Aphasia Evaluation; Boston Naming Test; Controlled Word Assoication; Category Naming; Mattis Dementia Rating Scale and Wechsler Adult Intelligence Scale.	3	Composite cognition (computed via sum of scores from all tests).	Significant interaction between Mediterranean diet and time among individuals without dementia (N = 1964), suggesting better adherence to Mediterranean diet is associated with reduced rate of cognitive decline, (per additional unit of adherence to MeDi equal to = β = 0.003; P = 0.047).	Cohort, age, gender, ethnicity, education, APOE genotype, caloric intake, smoking, comorbidity index and BMI.
Shakersain et al., (2018) [77] *SNAC-K,*; Sweden.	6	2,223	Dementia-free individuals aged ≥60, living at home or institutions on the island of Kungsholmen (60.8% female).	≥60	98-item FFQ; 1 time point.	1. MIND, Morris et al. (2015).<br><br>2. Mediterranean Diet, Panagiotakos et al. (2007).	MMSE	3	MMSE	Significant association between adherence to MIND, Mediterranean diet, Baltic sea index and Nordic prudent DP and change in MMSE score over 6 years	Total calorie intake, age, sex, education, civil status, physical activity, smoking, body mass index, vitamin/mineral supplement intake, vascular disorders, diabetes, cancer, depression, APOE E4, and dietary components other than those included in each dietary index.

						3. DASH, from Smith et al. (2010)<br><br>4. Baltic sea diet, from Kanerva et al. (2014).<br><br>5. Nordic prudent DP, (A-posteriori via EFA).				(β, T_3 vs T_1 = 0.126; 95% CI: 0.064, 0.188; P < 0.001; β, T_3 vs T_1 = 0.099; 95% CI: 0.036, 0.163; P = 0.002; β, continuous = 0.004; 95% CI: 0.000, 0.008; P = 0.049; β, $T_{3 \text{ vs }} T_1$ = 0.239; 95% CI: 0.176, 0.302; P = <0.001, respectively).<br>No significant association between adherence to DASH and change in MMSE score over 6 years.	
Shakersain et al., (2016) [6]; *SNAC-K;* Sweden.	6	2,223	Dementia-free individuals aged ≥60, living at home or institutions on the island of Kungsholmen (60.8% female).	70.6 ± 8.9	98-item FFQ; 1 time point.	1. Nordic prudent DP, (A-posteriori via EFA).<br><br>2. Nordic western DP, (A-posteriori via EFA).	MMSE	3	MMSE	Significant association between adherence to both Nordic prudent DP and western DP and change in MMSE score over 6 years (β, $Q_{5 \text{ vs }} Q_1$ = 0.106; 95% CI: 0.024, 0.189; P = 0.011; β, $Q_{5 \text{ vs }} Q_1$ = -0.156; 95% CI: -0.240, -0.073; P = < 0.001, respectively).	Age, sex, education, total caloric intake, civil status, smoking, physical activity, multivitamin/supplement intakes, vascular disorders, diabetes, cancer, depression, APOE E4, other dietary pattern score.
Shi et al., (2019) [79]; *China Health and Nutrition*	9	4,852	Individuals living within selected areas of China aged 55 and older	≥55	3-day food diary;	1. Iron-related DP, (A-	TICS-m; Immediate recall of a 10-word list and delayed recall of a 10-word	≥1-4	Global cognition (computed via sum of	Significant association between adherence to iron-	Age, gender, total caloric intake, fat intake, alcohol drinking, income, urbanicity, education,

Survey; China .			at follow-ups (61.1% female).		Multiple time points.	posteriori via RRR).	list; Counting backward from 20; Serial 7 subtraction.		scores from all tests).	related DP and change in global cognition across 9 years (β, $Q_{4\ vs}\ Q_1$ = -0.79, 95% CI: -1.25, -0.32; P = <0.001).	physical activity, BMI, hypertension.
Smyth et al., (2015) [80]; *ONTARGET and TRANSCEND;* Cross-country.	Median follow-up for participants with 2 MMSE's = 4.9 (4.4-5.0) years	27,860	Individuals aged 55 years and older with a history of one or more of coronary, cerebral, or peripheral artery disease, or high-risk diabetes mellitus (29.2% female).	56	Qualitative 20-item FFQ; 1 time point.	1. Modified Alternative Healthy Eating Index (mAHEI), from Dehghan et al. (2012).	MMSE	3	Cognitive decline (defined as a decrease of ≥3 points in MMSE score at any point in follow up, computed by subtracting score at last follow up from baseline score).	Significant association between MA-HEI and change in cognitive decline (HR, $Q_{5\ vs}\ Q_1$: 0.76; 95% CI: 0.66, 0.86; P for trend <0.01).	Age, education, sex, trial enrollment, treatment allocation, geographical region, baseline MMSE, systolic blood pressure, stroke/TIA, diabetes, myocardial infarction, microalbuminuria, macroalbuminuaria, serum creatinine, statin therapy, beta-blocker therapy, antithrombotic use, smoking, BMI, depression.
Tsai, (2014) [85]; *Taiwan Longitudinal Study of Ageing (TLSA)*; Taiwan.	8	2,988	Individuals aged ≥65 in 1999 (45.7% female).	73 ± 6	Brief FFQ from 1999 survey of TLSA; 1 time point.	1. Western DP, (Authors' own scoring system).<br>2. Traditional DP, (Authors' own scoring system).<br>3. Healthy DP, (Authors' own scoring system).	Short portable mental state questionnaire (SPMSQ).	3	Cognitive decline (defined as SPMSQ score at 1999 >2 points and SPMSQ score decreased ≥2 points over 4 and 8 years).	Significant negative association between adherence to the western DP and rate of cognitive decline across 8 years (OR = 4.35; 95% CI: 1.52, 12.50; P = 0.01. However, no significant association was found between either the traditional DP or healthy DP and rate of cognitive	Sex, age, years of formal education, lifestyle factors and ADL score at 1999.

										decline across 8 years.	
Vercambre et al., (2012) [86]; *Women's Antioxidant Cardiovascular Study (WACS)*; USA.	5.4 Range 4.1-6.1 y	2,504	Participants from WACS (female healthcare professionals) who participated in an RCT of vitamin E, vitamin C, and beta carotene supplementation for secondary prevention of CVD (100% female).	≥65	116-item semi-quantitative FFQ; 1 time point.	1. Mediterranean diet, Adapted from both Trichopoulou et al. (2003) and Panagiotakos et al. (2007).	Telephone Interview of Cognitive Status (TICS); TICS 10-word list; Telephone adaptation of MMSE; EBMT; Category Fluency Test.	4	Global cognition (computed via sum of scores from all tests); TICS score.	No significant association between adherence to the Mediterranean diet and change in global cognition or TICS score across 5 years.	Age, education, total caloric intake, marital status, multivitamin use, physical activity, BMI, smoking, postmenopausal hormone therapy, aspirin use, NSAID use, depression, cardiovascular profile (myocardial infarction, stroke, revascularisation procedures, angina, cerebral ischaemia), diabetes, hypertension, hyperlipidaemia, randomisation assignment for vitamin E, C, beta carotene and folate (placebo, active or not assigned).
Shatenstein et al., (2012) [78]; *NuAge Study;* Canada.	3	1,488	Community-dwelling older adults aged 67-84 (52.6% female).	Males: 74.05 ± 4.09<br><br>Females: 74.36 ± 4.21	78-item validated – semi-quantitative FFQ; 1 time point.	1. Canadian Healthy Eating Index (C-HEI) from Shatenstein et al. (2005).	3MS	4	Modified Mini-Mental State Examination (3MS)	No significant association between C-HEI and change in 3MS score across 3 years.	Age, sex, education, physical activity, family income, alcohol, waist circumference, depression, mental and total functional autonomy, vitamin/supplement use, total C-HEI, total caloric intake.
Wengreen et al., (2013) [88]; *CACHE County Study on Memory,*	10.6	3,580	All residents of Cache County aged 65 or above in 1995 (baseline) (38-54.9% male).	≥65	142-item FFQ; 1 time point.	1. Mediterranean diet, adapted from Trichopoulou et al. (2003).	3MS	4	3MS	Significant association between adherence to Mediterranean diet and DASH diet and change in	Age, sex, education, BMI, frequency of physical activity, multivitamin use, mineral supplement use, alcohol and smoking status, diabetes history, heart attack and stroke.

Health and Ageing; USA.						2. DASH, from Fung et al. (2008).				3MS scores across 11 years (Mean difference, $Q_{5 \text{ vs } Q_1}$ = 0.94; S.E: 0.29; P = 0.0014; Mean difference, $Q_{5 \text{ vs } Q_1}$ = 0.97; S.E: 0.29; P = 0.0009), respectively.	
Wengreen et al., (2009) [89]; *CACHE County Study on Memory, Health and Ageing*; USA.	11	3,634	All residents of Cache County aged 65 or above in 1995 (baseline) (55-59% male).	74.6	142-item FFQ; 1 time point.	1. Recommended food score (RFS) according to diet guidelines for Americans, adapted from Kant et al., (2000) and modified by Kaluza et al. (2009).	3MS	4	3MS	Significant association between adherence to RFS and change in 3MS across 11 years (β, $Q_{5 \text{ vs } Q_1}$ = 3.41; S.E: 0.79; P = 0.0013).	Age, sex, education, BMI, apoe e4, smoking, alcohol use, physical activity, myocardial infarction, stroke, diabetes, multivitamin/mineral use, total caloric intake, time x time, interactions between time and increasing quintile of RFS.
Parrott et al., (2013) [71]; *NuAge Study;* Canada.	3	1,099	Community-dwelling older adults aged 67-84, without cognitive impairment at baseline (49.4% female).	74.1 ± 4.1	78-item validated semi-quantitative FFQ; 1 time point.	1. Prudent pattern, (A-posteriori via PCA).<br><br>2. Western pattern, (A-posteriori via PCA).	3MS	4	3MS	No significant association between prudent DP and change in 3MS score across 3 years overall. But association was significant among those with low composite socio-economic position (B = 0.25; 95% CI: 0.0094, 0.50; P = 0.042).<br><br>No significant association between western DP and change in 3MS score across 3	Age, sex, energy intake, education, physical activity, medication usage, vitamin supplement usage, natural health product usage, social engagement, depression, perceived health status, smoking, waist circumference, BMI, hypertension, T2DM, systolic blood pressure, income, occupation and their interactions with time.

										years (B = -0.081; 95% CI: -0.21, 0.050; P = 0.23). Association was significant among those with low education attainment (B = -0.23; 95% CI: -0.43, -0.032; P = 0.023).	
Granic et al., (2016) [62]; *Newcastle 85+ Study;* UK.	5	851	Participants must have been born in 1921 and recruited through GPs within Newcastle and North Tyneside (61.8% female).	≥85	Validated 24-hr diet recall; 1 time point.	1. High red meat, (A-posteriori via cluster analysis).<br>2. Low meat, (A-posteriori via cluster analysis).<br>3. High butter, (A-posteriori via cluster analysis).	Standardised MMSE (SMMSE); Simple reaction time; Choice reaction time; Digit vigilance task.	4	Global cognition (computed via Standardised MMSE).	Significant association between low meat DP and higher SMMSE scores cross-sectionally at baseline and across follow-ups, compared to individuals with individuals in high red meat DP and high butter DP. High red meat DP and higher butter DP were associated with worse SMMSE performance. No significant differences in longitudinal decline rate dependent on DP over 5 years.	Age, baseline diet pattern and interaction term, sex, education, marital status, social class, smoking, physical activity and diet change in past year, multi-morbidity, BMI and APOE genotype.
Koyama et al., (2014) [64]; *ABC Study;* USA (Koyama et al. 2015).	7.9 ± 0.1	2,326	Adult participants aged 70-79, community-dwelling from a random sample of	74.6 ± 2.9	Interviewer administered 108-item FFQ; 1 time point.	1. Mediterranean diet, from Panagiotakos et al. (2007).	3MS	5	3MS	All subjects were analysed by racial status. Significant association between adherence to Mediterranean	Age, sex, education, BMI, smoking, physical activity, depression, diabetes, SES, APOE e4.

			selected areas (51.3% female).							diet and trajectory of 3MS score across 7.9 years among blacks (N = 874) (Mean difference in slope of 3MS score per year, T_3 vs $T_{1\text{-}2}$ = 0.25 points/year; 95% CI: 0.08, 0.42; P = 0.005). No significant association between adherence to Mediterranean diet and trajectory of 3MS score across 7.9 years among whites (N = 1452).	
Tanaka et al., (2018) [8]; *InCHIANTI study;* Italy.	10.1	832	Participants were older individuals (≥65 at baseline) residing within Chianti region, Italy (56.5% female).	75.4 ± 7.6	Validated FFQ; 1 time point.	1. Mediterranean diet, from Trichopoulou et al. (2003).	MMSE	5	Cognitive decline (defined as a 5-point decrease in MMSE score at any of the follow-up visits); MMSE.	Significant association between adherence to the Mediterranean diet, risk of cognitive decline (HR, $T_{3\text{ vs }}T_1$ = 0.59, 95% CI: 0.39, 0.88; P = 0.011) and change in MMSE across 10-year period (average follow up) (β, $T_{3\text{ vs }}T_1$ = 0.346, S.E. 0.077; P < 0.001).	Age, sex, study site, chronic diseases, education, total caloric intake, physical activity, BMI, Apoe E4, CRP IL-6, plasma omega 3, plasma omega 6, beta carotene, plasma alpha tocopherol.
Tangney et al., (2011) [82]; *CHAP;* USA.	7.6	3,790	Participants were older individuals (≥65 at baseline) residing within	75.4 ± 76.2	139-item FFQ; 1 time point.	1. Mediterranean diet, from	MMSE; Immediate and delayed recalls of the East Boston Memory Test;	5	Global cognition (computed via sum of	Significant association between adherence to both the Mediterranean	Age, sex, race, education, participation in cognitive activities, total caloric intake, interaction

			Chicago, USA (61.7% female).			Panagiotakos et al. (2007).<br><br>2. Mediterranean Diet Wine Score, Adapted from Panagiotakos et al., (2007).<br><br>3. Healthy Eating Index Score (HEI-2005), from Guenther, Reedy, and Krebs-Smith (2008).	Symbol digit modalities test.		scores from all tests).	diet and Mediterranean diet wine score and rate of change in global cognition across 7.6 years (β, continuous = 0.0014; S.E.E = 0.0004; P = 0.0004; β, continuous = 0.0014; S.E.E = 0.0004; P = 0.0009), respectively. However no significant association between adherence to the HEI score and rate of change in global cognitive scores across 7.6 years.	between time and each dietary quality score.
Tangney et al., (2014) [83]; *MAP*; USA.	4.1 Range (1-10 y)	826	Older individuals living in retirement communities and subsidised housing (26% male).	81.5 ± 7.1	144-item semi-quantitative validated FFQ; 1 time point.	1. DASH diet, from Folsom, Parker, and Harnack (2007).<br><br>2. Mediterranean diet, from Panagiotakos et al. (2007).	Immediate and delayed logical memory; Consortium to Establish a Registry for Alzheimer's Disease [CERAD] immediate and delayed word list recall; CERAD word list recognition; East Boston Story immediate, and delayed; Verbal fluency from CERAD; 15-item version of the Boston Naming Test; 15-item	Up to 10	Global cognition (computed via sum of scores from all tests).	Significant association between both adherence to DASH and Mediterranean diet and change in global cognition scores across 4.1 years (β, $T_{3\ vs}\ T_1$ = 0.022; S.EE = 0.011; P = 0.04; β, $T_{3\ vs}\ T_1$ = 0.034; S.EE = 0.012; P = 0.003, respectively).	Terms for the dietary pattern, age, sex, education, participation in cognitive activites, total caloric intake, time, and the interaction between time and each covariate, physical activity, Apoe E4, depression, stroke.

							reading test; Digit Span subset Forward; Digit Span subset Backward; Digit Ordering; Oral version of the Symbol Digit Modalities Test; Number Comparison; 2 indices from the modified Stroop Neuropsychological Screening; 15-item Judgment of Line Orientation; 16-item of Standard Progressive Matrices.				
Richard et al., (2018) [73]; *Rancho Bernardo Study*; USA.	9 ± 7.7	1,499	Participants were predominantly white, middle to upper class adults aged 50+ (57-59% female).	73.2 ± 9.2	153-item Willett FFQ; 1 time point.	1. Alternative Modified Mediterranean Diet, Adapted from Trichopoulou et al. (2003).<br>2. AHEI-2010, Chiuve et al. (2012).<br>3. Fortified cereals, (A-posteriori via PCA).<br>4. Fruits and vegetables, (A-posteriori via PCA).<br>5. Animal fat/vit b12, (A-	Mini mental state examination (MMSE); Trail-making Test Part B; Category fluency; Buschke Selective Reminding task.	7	MMSE; Trails B; Verbal Fluency; Buschke Total Recall.	No significant associations found between any DP explored and change in MMSE, Trails B, Verbal fluency or Buschke total recall across 9 years when comparing T_3 vs T_1 of each DP.	Age, sex, education, total caloric intake, time, time squared, smoking, exercise, alcohol consumption.

						posteriori via PCA).<br><br>6. Dairy, (A-posteriori via PCA).<br><br>7. Plant PUFA/vit E, (A-posteriori via PCA).<br><br>8. Sugar/ low protein, (A-posteriori via PCA).					
Agarwal et al., (2020) [53] *CHAP*, USA.	6.3 ± 2.8	5,001	Participants were older individuals (≥65 at baseline) residing within Chicago, USA.	74.05 ± 6.0	144-item semi-quantitative validated FFQ; 1 time point.	1. Mediterranean diet, Panagiotakos et al., (2007) [1].<br><br>2. Western (a-posteriori via PCA)	East Boston Story immediate and delayed recall; MMSE; Symbol digit modalities test.	Up to 10	Global cognition (computed via sum of scores from all tests).	1. Significant association between adherence to Mediterranean diet and change in global cognition scores across 6.3 years (β, $T_{3\ vs}\ T_1$ = 0.022; 95% CI = = 0.010, 0.033; P = 0.0003).<br><br>2. No significant association for either a Western DP (β, $T_{3\ vs}\ T_1$ = 0.0001; P = 0.96), or a healthy DP (β, $T_{3\ vs}\ T_1$ = 0.00001; P = 0.32) on global cognition.	Age, sex, race, education, physical activity late life cognitive activities, total caloric intake, diabetes, hypertension, stroke, myocardial infarction.
Boumenna et al., (2022) [55], *Boston Puerto*	8	1,332 (n = 470 full data).	Middle-aged and older Puerto Rican adults living in mainland USA	57.2 ± 7.9	Validated FFQ; 3 time points.	1. MIND, Morris et al., (2015) [2].	MMSE; 16-word list learning test; Stroop test; Digit span forward and backward test;	3	Global cognition (computed via sum of	1. Significant positive association between adherence to	Age, sex, BMI, physical activity score, diabetes, hypertension, smoking, alcohol use, APOE E4 carrier, energy intake, job

Rican Health Study, USA.			(Boston) (70% female).				Verbal fluency; Clock drawing; Figure copying.		scores from all tests).	MIND and global cognition during follow up (β, Q_1 vs Q_5 = 0.0093; 95% CI = 0.035, 0.152; P = 0.019) but no association of MIND on rate of change in 8 year global cognitive trajectory.	complexity score, poverty index.
Chen et al., (2021) [56], *Sydney Memory and Ageing Study*, Australia.	6	1,037	Individuals aged 70-90 recruited from Sydney (55% female).	78.8 ± 4.8	Validated 74-item FFQ; 1 time point.	1. Australian Dietary Guideline Index-2013.	Digit symbol coding; Trail making test A; Boston naming test; Animals; Controlled Oral word Association test; Trail making test B; Block design test; Logical memory story A delayed recall; Rey Auditory Verbal Learning Test; Benton Visual Retention Test Recognition).	4	Global cognition (computed via average of cognitive domain scores).	1. No association between adherence to the DGI-2013 and change in global cognition across 6 years (β = 0.000; 95 % CI: −0.007, 0.007; P = 0.41).	Age, sex, education, non-English speaking background, physical activity, ethnicity, BMI, hypertension, diabetes, hypercholesterolaemia, stroke/transient ischaemic attack, smoking, depression, ApoE E4.
Chen et al., (2022) [57], *Sydney Memory and Ageing Study*, Australia.	6	1,037	Individuals aged 70-90 recruited from Sydney (55% female).	78.8 ± 4.8	Validated 74-item FFQ; 1 time point.	1. Mediterranean diet, Panagiotakos et al., (2007) [1].<br><br>2. Mediterranean diet, Trichopoulou et al., (2003) [3].	Digit symbol coding; Trail making test A; Boston naming test; Animals; Controlled Oral word Association test; Trail making test B; Block design test; Logical memory story A delayed recall; Rey Auditory Verbal Learning Test;	4	Global cognition (computed via average of cognitive domain scores).	1. No association between adherence to the Mediterranean diet and change in global cognition across 6 years (β = 0.005; 95 % CI: -0.018, 0.028; P = 0.690.<br><br>2. No association between adherence to the	Age, sex, education, non-English speaking background, physical activity, ethnicity, BMI, hypertension, diabetes, hypercholesterolaemia, stroke/transient ischaemic attack, smoking, depression, ApoE E4.

						3. DASH DP, Berendsen et al., (2017).	Benton Visual Retention Test Recognition).			Mediterranean diet and change in global cognition across 6 years (β = -0.026; 95 % CI: −0.093, 0.041; P = 0.446).<br><br>3. No association between adherence to the DASH DP and change in global cognition across 6 years (β = 0.008; 95 % CI: −0.019, 0.035; P = 0.570).	
Corley and Deary (2020) [58], *Lothian Birth Cohort*, UK.	12.53 ± 0.5	863	Adults born in 1936 who are community dwelling and free from dementia at baseline (50.2% male).	69.5 ± 0.8	168-item FFQ; 1 time point.	1. Mediterranean-style DP, (a-posteriori via PCA).<br><br>2. Scottish traditional DP, (a-posteriori via PCA)	Wechsler Adult Intelligence Scale III-UK edition; Wechsler Memory Scale III-UK edition.	5	Global cognition (computed via average of scores from 13 cognitive tests).	1. No association between adherence to the Mediterranean style DP and global cognitive change across 12 years (β = -0.002, S.E = 0.001; P = 0.17).<br><br>2. No association between adherence to the traditional DP and global cognitive change across 12 years (β = -0.00, S.E. = 0.001; P = 0.88).	Age, sex, diet score, 11 year intelligence quotient, marital status, socio-economic status, physical activity, smoking status, ApoE e4.

Keenan et al., (2020) [63], *Age-related Eye Disease Study (AREDS 2 alone as cognitive reassessment not performed in AREDS).*	6.5 ± 3.8	3,326	Adults from AREDS2 with no age related macular degeneration (AMD) to unilateral AMD (57.8% female).	72.9 ± 7.7	130-item semi-quantitative block FFQ; 1 time point.	1. Mediterranean DP, Lopez-Garcia et al., (2014).	TICS-M; TICS-M recall; Animal category; Letter fluency; Alternating fluency; Logical memory part I & part II; Digits backwards.	4	Composite score (sum of overall test battery).	1. No significant association (P for trend = 0.91), between adherence to the Mediterranean diet and longitudinal change in composite cognitive score at 2 year follow-up (Mean difference, T_3 vs T_1 = 1.6), 4 year follow-up (Mean difference, T_3 vs T_1 = 1.4), or 10 year follow up (Mean difference, T_3 vs T_1 = 1.2).	Age, sex, race, smoking, diabetes, hypertension, baseline depression score, calorie intake and years from baseline.
Lotan et al., (2022) [65]; *Israel Diabetes and Cognitive Decline (IDCD)*, Israel.	4.1 ± 2.1	960	Individuals aged 65 and over living in central Israel with T2DM (42.7% female).	71.6 ± 4.6	Semi quantitative 126-item FFQ; 1 time point.	1. Mediterranean DP, Panagiotakos et al., (2007) [1].<br><br>2. MIND DP, Morris et al., (2015) [2].<br><br>3. DASH DP, Folsom et al., (2007) [5].	MMSE; Immediate recall; Delayed recall; Word recognition from ADAS-COG battery; Shape Cancellation; Digit Span Forward and Backward; Similarities; Animal Fluency; Boston Naming Test; Trail Making Test A & B; Praxis from ADAS-COG; Digit symbol substitution test.	≥1	Global cognition (sum of average domain scores).	1. Positive association between Mediterranean diet and change in global cognition across follow-up (β = 0.00323; S.E. = 0.00123; P = 0.008) in fully adjusted models.<br><br>2. No association between adherence to MIND DP and change in global cognition across follow-up in fully adjusted model (β = 0.00604; S.E. = 0.00354; P = 0.087).	Age, sex, education, daily calories, duration of T2D at baseline, cholesterol, creatinine, HbA1c, triglyceride, systolic blood pressure, diastolic blood pressure, body mass index, diabetic medication and physical activity.

										3. No association between adherence to DASH DP and change in global cognition across follow-up in any model (β = 0.00342; S.E. = 0.00536; P = 0.524).	
Mueller et al., (2020) [67], *WRAP*, USA.	6.3	828	Participants with parental history of late-onset AD (67.6% female).	57.4 ± 6.4	Self-administered MIND screening questionnaire; 1 time point.	1. MIND DP, Morris et al., (2015).	Rey Auditory Verbal Learning Test; Delayed recall of stories A and B from Wechsler Memory Scale; Digit Substitution Test; MMSE.	2-6	Preclinical Alzheimer's Cognitive Composite 4 (PACC4) (Composite of tests).	No association between adherence to MIND DP and change in PACC4 scores (β = 0.018; 95% CI = -0.66, 0.54).	Age, sex, APOE E4 risk score, wide range achievement reading test score (proxy for education).
Muñoz-García et al., (2021) [68], *SUN Study*, Spain.	6	803	Participants were university graduates from Navarra University (70% male).	67 ± 5	136-item semi-quantitative FFQ; 1 time point.	1. Western DP (a posteriori via PCA).<br>2. Mediterranean-style DP (a posteriori via PCA).	Spanish version of the modified Telephone Interview for Cognitive Status (STICS-m).	3	STICS-m	1. Negative association between adherence to the Western DP and 6-year change in STICS-m (β, T_3 vs T_1 = -0.80; 95% CI = -1.51, -0.08; P = 0.03).<br>2. Positive association between adherence to the Mediterranean-style DP and 6 year change in STICS-m (β, T_3 vs T_1 = 0.71; 95% CI = 0.15, 1.26; P = 0.012).	Age, sex, follow-up time until baselines STICS-m, years of university education, APOE E4, smoking and number of package-years, total caloric intake, physical activity, BMI, alcohol intake, depression, hypertension, high cholesterol, low HDL cholesterol, cardiovascular disease, T2DM, atrial fibrillation.

Nooyens et al., (2021) [69], *Doetinchem Cohort Study*, Netherlands.	15 (median)	3,719	Adults aged ≥45 years old at baseline originally recruited in 1987-91 (51% women).	56 ± 7	178-item semi-quantitative validated FFQ; ≥1-2 time points.	1. Mediterranean diet, Trichopoulou et al., (2003) [3].<br><br>2. HDI DP, Amine et al., (2002).<br><br>3. Modified Dutch Healthy Diet 2015 Index (mDHD15) (adapted), Looman et al., (2017).	15-word verbal learning test; Stroop colour word test; Verbal fluency test (animal naming); Letter digit substitution test.	5	Global cognition (computed via sum of all test scores).	1. Positive association between adherence to the Mediterranean DP and difference in global cognition between 55-75 years (Mean difference, T_3 vs T_1 = 7.4%, 95% CI = 1.0, 14.9; P = <0.05).<br><br>2. Positive association between adherence to the HDI DP and difference in global cognition across follow-up (Mean difference, T_3 vs T_1 = 6.5%, 95% CI = 0.3, 13.7; P = <0.05).<br><br>3. Positive association between adherence to the mDHD15 DP and difference in global cognition across follow-up (Mean difference, T_3 vs T_1 = 6.5%, 95% CI = 0.6, 13.6; P = <0.05).	Age, Tertiles x age, sex, education, apoe4 genotype, physical activity, smoking, marital status, energy intake, vitality, mental health.
Soldevila-Domenech et al., (2021) [81], *PREDIMED-*	3	487	Subset of PREDIMED-Plus. Overweight/obese adults aged 55-75	65.2 ± 4.7	17-item questionnaire of adherence to	1. Mediterranean diet (energy-restricted),	RAVTL immediate recall; RAVTL delayed recall; RCFT immediate	3	Global cognition (as latent variable).	1. No significant association between adherence to	Gender, age, years of education, baseline weight, IQ, use of treatment for high cholesterol, use of

Plus-Cognition, Spain.			with ≥3 criteria for metabolic syndrome (49.5% male).		Mediterranean diet; 1-3 time points.	Schröder et al., (2011) (137).	recall; RCFT delayed recall; RCFT recognition; RCFT copy; SDMT; Stroop interference; CPT-omission errors; CPT-commission errors; CPT-HRT; and IGT.			Mediterranean DP at T1 and change in global cognition after 3 years (Standardised estimate = 0.040; P = >0.05). N.B. Positive association between Mediterranean DP at T1 and change in memory after 3 years (Standardised estimate = 0.13; P = <0.05)	tranquilizers or sedatives, prediabetes, diabetes and current smoking status.
Tong et al., (2021) [84], *Singapore Chinese Health Study*, Singapore.	19.7	14,683	Adult individuals living in Singapore in government-built housing estates (59.1% women).	72.9	165-item FFQ; Modified 21-item dietary screener; 1 time point each.	1. DASH (adapted), Fung et al., (2008) [4].	Singapore-modified version of the MMSE (S-MMSE)	3 time points.	Cognitive impairment (via cut-offs on S-MMSE)	1. Positive association between adherence to DASH and reduced odds of cognitive impairment at follow-up (OR = 0.50; 95% CI = 0.42, 0.59; P = <0.001).	Age, sex, year of baseline interview, dialect, education level, marital status, smoking, sleep, BMI, new cases of hypertension, coronary artery disease, stroke, diabetes, alcohol drinking and weekly activities.
Vu et al., (2022) [87]; (*CHAP only for cognitive decline), MAP & WHIMS*, USA.	Dependent on cohort.	CHAP = 2,449.	Samples from CHAP, MAP and WHIMS. Data presented is from CHAP in relation to MIND for this article. Data from MAP presented in other study with larger sample size.	CHAP white = 72.2-74; CHAP black =71.1-71.7.	CHAP = 139-item FFQ. FFQs conducted at multiple timepoints but analysis used data from one time point.	1. MIND, Morris et al., (2015) [2].	MMSE; Immediate and delayed recalls of the East Boston Memory Test; Symbol digit modalities test.	5	Global cognition (computed via sum of scores from all tests).	1. No association between adherence to MIND DP and cognitive decline across follow-up in either white or black individuals in CHAP, respectively (β, T_3 vs T_1 = -0.008; 95% CI = -0.01, 0.01; P = 0.89; β, T_3 vs T_1 = -	Age, sex, race, education, participation in cognitive activities, total caloric intake, interaction between time and each dietary quality score.

										0.003; 95% CI = -0.01, 0.01; P = 0.51).	

<u>Supplementary Table S5.</u> <u>Overview of prospective studies examining associations between any mild or major NCD and DPs</u>

Authors, year, *study name*, country	Study characteristics				Diet measures		Results			
	Follow-up time (years)± SD	N =	Population characteristics	Mean age at baseline, y	Food intake assessment; time point(s) assessed	DP(s) examined, (scoring reference or a-posteriori)	Diagnosis of outcome(s)	Primary outcome(s) of interest	Main findings from primary outcome of interest	Covariates
Akbaraly et al., (2019) [10]; *Whitehall II*; UK.	24.8	8,225	Civil servants aged 35-55 at baseline (30.9% female).	50.2 ± 6.1	127-item FFQ; 3 time point(s).	1. AHEI from Akbaraly et al., (2019).<br><br>2. Healthy DP, (a-posteriori via PCA).<br><br>3. Western DP, (a-posteriori via PCA).	Incident Dementia; International Statistical Classification of Diseases and Related Health Problems, 10th Revision codes, F00, F01, F02, F03, F05.1,G30, and G31.0.	Incident Dementia	1. No significant association between risk of incident dementia and adherence to the AHEI (adjusted HRs for dementia per 1-SD (10-point) = 0.97: 95% CI: 0.87, 1.08), in 1997-1999 (HR = 0.97; 95% CI: 0.83, 1.12) or in 2002-2004 (HR = 0.87; 95% CI: 0.75, 1.00).<br><br>2. No significant association between risk of incident dementia and adherence to the healthy DP (adjusted HRs for dementia per 1-SD increase = 0.93: 95% CI: 0.83, 1.05), in 1997-1999 (HR = 0.86; 95% CI: 0.72, 1.02) or in 2002-2004 (HR = 0.90; 95% CI: 0.76, 1.07).<br><br>3. No significant association between risk of incident dementia and adherence to the western DP in 1991-1993 (adjusted HRs for dementia per	Age, sex, race/ethnicity, total energy intake, education level, occupational position, marital status, smoking status, physical activity level, alcohol consumption, hypertension, type 2 diabetes, body mass index, dyslipidemia, depressive symptoms, coronary heart disease or stroke, and use of any cardiovascular disease medication.

									1-SD increase = 0.99: 95% CI: 0.83, 1.17), in 1997-1999 (HR = 1.03; 95% CI: 0.82, 1.30) or in 2002-2004 (HR = 0.89; 95% CI: 0.71, 1.12).	
Cherbuin & Anstey, (2011) [11]; *PATH Study;* Australia.	4	1,528	Community dwelling, middle-aged adults (51.2% female).	60-64	183-item FFQ; 1 time point.	1. Mediterranean DP, from Trichopoulou et al. (2003).	Dementia; DSM-IV. MCI; International Consensus Criteria, CDR scale.	MCI; Any mild cognitive disorder.	1. No significant association between adherence to MeDi (measured via continuous MeDi score) and risk of MCI (OR = 1.41; 95% CI: 0.95, 2.10; P = 0.087), or any mild cognitive disorder (OR = 1.20; 95% CI: 0.98, 1.47; P = 0.079).	Age, sex, education, Apoe e4, BMI, physical activity, stroke, diabetes, hypertension, total caloric intake.
Chuang et al., (2019) [109]; *NAHSIT Study;* Taiwan	11.04	1,413	Community-dwelling Taiwanese nationals aged 65 or above (49.3% female).	73	79-item FFQ and 16-item FFQ; 1 time point.	1. NAHSIT cognition-related DP, (A-posteriori via RRR).	Surveillance of national health insurance database (ICD-9).	Dementia	1. No significant association between adherence to cognition-related DP and risk of dementia (HR, T_3 vs T_1: 0.90; 95% CI:0.66, 1.32; P = 0.501).	Age, education, baseline cognition, BMI, stroke, blood pressure, inflammation status and stroke occurrence during study period.
Dearborn-Tomazos et al., (2019) [59]; *Atherosclerosis Risk in Communities Study (ARIC);* USA.	20	13,588	Community-dwelling individuals aged 45-64 years old and living in selected communities (55.8% female).	54.6 ± 5.7	66-item FFQ; 1 time point.	1. Western DP, (A-posteriori via PCA).<br>2. Prudent DP, (A-posteriori via PCA).	Established protocol including telephone interviews of participants/informants or hospital discharge (ICD-9 or ICD-10) codes and death certificate surveillance.	Incident Dementia.	1. No significant association between adherence to the Western DP and risk of incident dementia (HR, T_3 vs T_1: 1.06; 95% CI: 0.92, 1.22).<br>2. No significant association between adherence to the Prudent DP and risk of incident dementia (HR, T_3 vs T_1: 0.99; 95% CI: 0.88, 1.12).	Age, sex, education, race–field center, and total energy intake, APOE ε4 status, alcohol use history, smoking history, activity level, body mass index, total cholesterol, prevalent coronary heart disease, history of hypertension, diabetes, and stroke.
Feart et al., (2009) [60]; *Three City Study;* France.	5	1,410	Community-dwelling individuals aged 65 or older from electoral rolls (57.8-60.1% female).	75.9	40-item FFQ and 24-hour diet recall; 1 time point.	1. Mediterranean DP, Trichopoulou et al., (2003).	Dementia and AD; DSM-IV.	Dementia; Alzheimer's Disease	1. No significant association between adherence to the Mediterranean dietary pattern and risk of incident dementia (HR, T_3 vs T_1 = 1.12; 95% CI: 0.6, 2.10; P = .72). No significant association between adherence to the Mediterranean dietary pattern and risk of Alzheimer's	Age, sex, education, marital status, energy intake, physical activity, ≥5 medications per day, APOE genotype, BMI, depression score (CES-D), hypertension, hypercholesterolemia, diabetes, tobacco use,

									Disease (HR, T_3 vs T_1 = 0.86; 95% CI: 0.39, 1.88; P = 0.72).	stroke, and their interaction with time.
Gu et al., (2010) [106]; *WHICAP;* USA.	3.96 ± 3	2,148	Community-dwelling individuals aged 65 or older residing in Northern Manhattan (68% female).	77.2 ± 6.6	Interviewer administered 61-item validated semi-quantitative FFQ; 1 time point.	1. 7 a-posteriori DPs, (A-posteriori via RRR).	Probable or possible Alzheimer disease (AD); National Institute of Neurological and Communicative Disorders and Stroke and the Alzheimer's Disease and Related Disorders Association (NINCDS-ADRDA).	Alzheimer's Disease	1. Significant association between adherence to DP2 (of the dietary patterns created A-posteriori) and decreased risk of AD (HR, T_3 vs T_1 = 0.62; 95% CI: 0.43,0.89; P = 0.01)	Recruitment cohort, age, sex, education, ethnicity, BMI, smoking, energy intake, alcohol intake, APOE status, Charlson Comorbidity Index 28 (Includes myocardial infarction, congestive heart failure, peripheral vascular disease, hypertension, chronic obstructive pulmonary disease, arthritis, gastrointestinal disease, mild liver disease, diabetes, chronic renal disease, and systemic malignancy from the initial visit).
Haring et al., (2016) [91]; *Women's Health Initiative Memory Study*; USA.	9.11	6,425	Women aged 65 or older recruited from Women's Health Initiative Hormone Trials (100% female).	65-79	122-item FFQ; 1 time point.	1. aMED DP, Adapted from Fung et al. (2009)<br><br>2. Healthy eating index-2010 DP (HEI-2010) from Guenther, Reedy, and Krebs-Smith (2008).<br><br>3. AHEI-2010 DP, from Chiuve et al. (2012).<br><br>4. DASH DP, from Fung et al. (2008).	Dementia and MCI; DSM-IV.	Probable dementia; MCI.	1. No significant association between adherence to the aMED and risk of probable dementia (HR, Q_5 vs Q_1 = 1.13; 95% CI: 0.79, 1.63; P = 0.46) or risk of MCI (HR, Q_5 vs Q_1 = 0.82; 95% CI: 0.59, 1.14; P = 0.08).<br><br>2. Significant association between adherence to the HEI-2010 and risk of probable dementia (HR, Q_5 vs Q_1 = 1.60; 95% CI: 1.10, 2.33; P = 0.02) but not with risk of MCI (HR, Q_5 vs Q_1 = 0.90; 95% CI: 0.66, 1.22; P = 0.43).<br><br>3. No significant association between adherence to the	Age, ethnicity, education, family income, BMI, smoking, HTN, physical activity, diabetes, depression, history of cardiovascular disease, HRT, modified MMSE score, total energy intake.

									AHEI-2010 and risk of probable dementia (HR, Q_5 vs Q_1 = 1.01; 95% CI: 0.71, 1.46; P = 0.71) or risk of MCI (HR, Q_5 vs Q_1 = 0.75; 95% CI: 0.54, 1.03; P = 0.10).<br><br>4. No significant association between adherence to DASH and risk of probable dementia (HR, Q_5 vs Q_1 = 1.28; 95% CI: 0.86, 1.91; P = 0.11) but it was significantly associated with risk of MCI (HR, Q_5 vs Q_1 = 0.72; 95% CI: 0.52, 1.02; P = 0.04).	
Hosking et al., (2019) [103]; *PATH Study;* Australia.	12	1,220	Community dwelling, middle-aged adults (42-60% female).	60-64	183-item FFQ; 1 time point.	1. Mediterranean DP, from Trichopoulou et al. (2003).<br><br>2. Mediterranean DP, Panagiotakos et al. (2007).<br><br>2. MIND DP, from Morris et al. (2015).	AD or vascular dementia (VaD); National Institute of Neurological Disorders criteria.<br>MCI; According to Winblad criteria (2004).	Cognitive impairment (defined as diagnoses of MCI or Dementia).	1. No significant association between adherence to Mediterranean diet (Trichopoulou et al., 2003), and odds of cognitive impairment (OR, T_3 vs T_1 = 1.30; 95% CI: 0.79, 2.15; P = 0.29).<br><br>2. No significant association between adherence to Mediterranean diet (Panagiotakos et al., 2007), and odds of cognitive impairment (OR, T_3 vs T_1 = 0.78; 95% CI: 0.44, 1.39; P = 0.40).<br><br>3. Significant association between adherence to MIND and reduced odds of cognitive impairment (OR, T_3 vs T_1 = 0.47; 95% CI: 0.25, 0.91; P = 0.03).	Energy intake, age, sex, APOE4 status, years of education, self-reported physical activity, mental activity participation, smoking, depression, BMI, heart disease, diabetes, stroke, hypertension.

Larsson et al., (2018) [120]; *SIMPLER*; Sweden.	12.3	28,775	Individuals aged 65-83 years old (46.6% female).	71.6 ± 4.5	96-item FFQ; 1 time point.	1. Modified DASH DP, from Fung et al. (2008).<br><br>2. Modified Mediterranean DP, from Trichopoulou et al. (2003).	Dementia; Swedish National Patient Register	Dementia	1. No significant association between adherence to modified DASH and risk of dementia (HR = 0.96; 95% CI: 0.87, 1.05).<br><br>2. No significant association between adherence to modified Mediterranean diet and risk of dementia (HR = 1.12; 95% CI: 0.96, 1.31).	Age, sex, education, BMI, hypertension, hypercholesterolemia, diabetes, and sleep duration.
Morris et al., (2015) [100]; *MAP*; USA .	4.5	923	Participants from retirement communities and senior public housing (22-26% male).	58-98	144-item Harvard FFQ; ≥2-10 time points.	1. MIND DP, from Morris et al. (2015).<br><br>2. DASH DP, from Folsom, Parker, and Harnack (2007).<br><br>3. Mediterranean DP, from Panagiotakos et al. (2007).	AD; National Institute of Neurological and Communicative Disorders and Stroke and the Alzheimer's Disease and Related Disorders Association (NINCDS-ADRDA).	Alzheimer's Disease (AD)	1. Significant association between adherence to MIND and reduced risk of AD (HR, T_3 vs T_1 = 0.48; 95% CI: 0.29, 0.79; P = 0.003).<br><br>2. No significant association between adherence to DASH and reduced risk of AD (HR, T_3 vs T_1 = 0.60; 95% CI: 0.38, 0.96; P = 0.06).<br><br>3. Significant association between adherence to Mediterranean diet and reduced risk of AD (HR, T_3 vs T_1 = 0.49; 95% CI: 0.29, 0.85; P = 0.01).	Age, sex, education, APOE4, participation in cognitively stimulating activities, physical activity, total energy intake, and cardiovascular conditions.
Olsson et al., (2015) [101]; *ULSAM;* Sweden.	12	1,038	Participants were males aged 70+ residing within the Uppsala municipality (100% male).	71 ± 0.6	7-day food record; 1 time point.	1. Healthy Diet Indicator (HDI) DP, from Sjögren et al. (2010) .<br><br>2. Modified Mediterranean DP, Trichopoulou et al. (2003).<br><br>3. Low Carb High Protein Score DP (LCHP),	Dementia; DSM-IV. AD; NINCDS-ADRDA.	All-type Dementia, Alzheimer's Disease (AD).	1. No significant association between adherence to HDI and risk of Dementia (HR, T_3 vs T_1 = 0.95; 95% CI: 0.46, 1.99; P = 0.92) or risk of AD, (HR, T_3 vs T_1 = 0.99; 95% CI: 0.39, 2.53; P = 0.99).<br><br>2. No significant association between adherence to modified Mediterranean diet and risk of Dementia (HR, T_3 vs T_1 = 0.85; 95% CI: 0.44, 1.62; P = 0.70) or	Energy, education, APOE ε4 allele, living alone, smoking, and physical activity.

						Trichopoulou et al. (2007).			risk of AD, (HR, T_3 vs T_1 = 0.99; 95% CI: 0.44, 2.26; P = 0.95).<br><br>3. No significant association between adherence to LCHP and risk of Dementia (HR, T_3 vs T_1 = 1.59; 95% CI: 0.85, 2.96; P = 0.14) or risk of AD, (HR, T_3 vs T_1 = 1.60; 95% CI: 0.74, 3.44; P = 0.21).	
Ozawa et al., (2013) [102]; Japan.	15 (median)	1,006	Participants were individuals aged 60-79 years who resided in Hisayama, Fukuoka metropolitan area (57% female).	60-79 years	70-item FFQ; 1 time point.	1. DP found to be related to intake of 7 nutrients (A-posteriori via RRR).	Dementia; DSM-IV . AD; NINCDS-ADRDA.	Dementia and Alzheimer's Disease (AD)	1. Significant association between adherence to the A-posteriori DP and risk of all-cause Dementia (HR, Q_4 vs Q_1 = 0.66; 95% CI: 0.46, 0.95; P = 0.02), risk of vascular Dementia, (HR, Q_4 vs Q_1 = 0.45; 95% CI: 0.22, 0.91; P = 0.02) but not with AD (HR, Q_4 vs Q_1 = 0.65; 95% CI: 0.40, 1.06; P = 0.17).	Education, diabetes, hypertension, total cholesterol, stroke, BMI, smoking, exercise and energy intake.
Scarmeas et al., (2009) [92]; *WHICAP*; USA.	4.5 ±2.7	2,364	Community-dwelling individuals aged 65 or older residing in Northern Manhattan (32% male).	76.9 ± 6.5	Interviewer administered 61-item validated semi-quantitative FFQ; 1 time point.	1. Mediterranean DP, from Trichopoulou et al. (2003).	MCI; Using criteria from definition by Petersen et al., 2001. AD; NINCDS-ADRDA.	MCI and conversion of MCI to AD	1. Borderline significant association between adherence to the Mediterranean diet and reduced risk of developing MCI (HR, T_3 vs T_1 = 0.72; 95% CI: 0.52, 1.00; P = 0.05), adherence to the Mediterranean diet was also significantly associated with reduced risk of conversion from MCI to AD (HR, Q_4 vs Q_1 = 0.52; 95% CI: 0.30, 0.91; P = 0.02).	Cohort, age, sex, ethnicity, education, APOE status, energy intake, BMI, duration between diet assessment and baseline diagnosis
Scarmeas et al., (2006) [76]; *WHICAP*; USA.	4 ± 3	2,258	Community-dwelling individuals aged 65 or older residing in Northern	77.2 ± 6.6	Interviewer administered 61-item validated semi-quantitative	1. Mediterranean DP, from Trichopoulou et al. (2003).	Dementia; DSM-IV. AD; NINCDS-ADRDA.	Alzheimer's Disease (AD)	1. Significant association between adherence to the Mediterranean diet and reduced risk of developing AD (HR, T_3 vs T_1 = 0.60; 95% CI: 0.42, 0.87; P = 0.007).	Cohort, age, sex, ethnicity, education, APOE status, energy intake, BMI, comorbidity index, smoking.

			Manhattan (32% male).		FFQ; 1 time point.					
Sindi et al., (2018) [96]; *CAIDE*; Finland.	21	341	Sub-sample of the CAIDE study including residents from Kuopio and Joensuu (61.1% female).	56	Diet questionnaire; 1 time point.	1. Healthy diet index DP, from Sindi et al., (2018).	Dementia; DSM. AD; NINCDS-ADRDA.	Incident dementia	1. Significant association between adherence to the healthy diet index and reduced odds of dementia (OR, T_3 vs T_1 = 0.41; 95% CI: 0.20, 0.85; P = <0.05).	Age, sex, education, cohort, healthy diet index, BMI, APOE4, physical activity, baseline cardio/cerebrovascular or respiratory conditions.
Tomata et al., (2016) [97]; *Ohsaki Cohort Study*; Japan.	5.7	14,402	Older individuals aged ≥65 years residing in Ohsaki City (44.8% male).	73.8 ± 5.9	39-item validated FFQ; 2 time points.	1. Japanese DP, (A-posteriori via PCA).<br><br>2. Animal food DP, (A-posteriori via PCA).<br><br>3. High dairy DP, (A-posteriori via PCA).	Incident Dementia; Criteria in long term care insurance system in Japan.	Incident dementia	1. Significant association between adherence to the Japanese DP and reduced risk of incident dementia (HR, T_3 vs T_1 = 0.80; 95% CI: 0.66, 0.97, P = 0.016).<br><br>2. No significant association between adherence to the Animal food DP and risk of incident dementia (HR, T_3 vs T_1 = 1.12; 95% CI: 0.92, 1.36, P = 0.216).<br><br>3. No significant association between adherence to the High dairy DP and risk of incident dementia (HR, T_3 vs T_1 = 0.97; 95% CI: 0.83, 1.15, P = 0.896).	Age, sex, stroke, myocardial infarction, hypertension, arthritis, osteoporosis, fracture, education level, smoking, alcohol, BMI, psychological distress score, time spent walking, motor function score, cognitive function score, number of remaining teeth, total caloric intake and protein intake.
Tsivgoulis et al., (2013) [105]; *Reasons for Geographic and Racial Differences in Stroke Study (REGARDS)*; USA.	4 ± 1.5	17,478	Individuals aged 45 years old or above, living within the Stroke belt in the USA, identifying as non-hispanic black or white (43% male).	64.4 ± 9.1	98-item FFQ; 1 time points.	1. Mediterranean DP; Trichopoulou et al. (2003).	Six-item screener (SIS).	Incident cognitive impairment (defined as shift from intact cognitive screening status at the first assessment (SIS score = 5–6) to impaired	1. Significant association between adherence to the Mediterranean diet and risk of incident cognitive impairment (after excluding cognitive assessments following incident stroke) (OR, High vs Low = 0.87; 95% CI: 0.76, 1.00; P = 0.0460).	Age, sex, ethnicity, region of residence, BMI, waist circumference, household income, education, smoking, alcohol use, physical activity, heart disease history, diabetes, atrial fibrillation, systolic blood pressure, diastolic blood pressure (DBP), high cholesterol, antihypertensive regimen (specific drug

								cognitive screening status at the latest available assessment (SIS score = ≤4).		classes), perceived general health status, and depressive symptoms.
Pearson et al., (2016) [104]; *REGARDS Study;* USA.	6.8	18,080	Individuals aged 45 years old or above, living within the Stroke belt in the USA, identifying as non-hispanic black or white (44.8-66.9% female).	64.8	98-item validated FFQ; 1 time point.	1. Convenience DP, (A-posteriori via PCA).<br><br>2. Plant-based DP, (A-posteriori via PCA).<br><br>3. Sweets/fats DP, (A-posteriori via PCA).<br><br>4. Southern DP, (A-posteriori via PCA).<br><br>5.Alcohol/salads DP, (A-posteriori via PCA).	Six-item screener (SIS).	Incident cognitive impairment (defined as shift from intact cognitive screening status at the first assessment (SIS score = 5–6) to impaired cognitive screening status at the latest available assessment (SIS score = ≤4).	1. No significant association between adherence to the Convenience DP and risk of incident cognitive impairment (OR, Q_5 vs Q_1 = 0.87; 95% CI: 0.70, 1.08; P for trend = 0.14).<br><br>2. No significant association between adherence to the Plant-based DP and risk of incident cognitive impairment (OR, Q_5 vs Q_1 = 0.89; 95% CI: 0.73, 1.10; P for trend = 0.23).<br><br>3. No significant association between adherence to the Sweets/fats DP and risk of incident cognitive impairment (OR, Q_5 vs Q_1 = 1.19; 95% CI: 0.95, 1.49; P for trend = 0.31).<br><br>4. Borderline significant association between adherence to the Southern DP and increased risk of incident cognitive impairment (OR, Q_5 vs Q_1 = 1.16; 95% CI: 0.93, 1.45; P for trend = 0.05).<br><br>5. Significant association between adherence to the Alcohol/salads DP and reduced risk of incident cognitive impairment (OR, Q_5 vs Q_1 =	Age, ethnicity, sex, region, total caloric intake, income, education, physical activity, smoking, BMI, hypertension, diabetes, CVD, depressive symptoms.

									0.68; 95% CI: 0.56, 0.84; P for trend = 0.0005).	
Andreu-Reinon et al., (2021) [108]; *EPIC-Spain;* Spain.	21.6 ± 3.4	16,160	Participants aged 30-70 at enrolment in EPIC-Spain studies in 1992-1996 (57.2-60.7% female).	30-70	Diet history questionnaire; 1 time point.	1. Mediterranean diet, from Trichopoulou et al. (2003).	Codes from ICD 9-10 (290, 331, F00-03, G30); codes from anatomical Therapeutic Chemical Classification (ATC) system; N06DA02, N06DA03, N06DDA04, and N06DX01); codes from international classification of primary care 2 (P20, P70, N29, and N99).	Incident dementia	1. Significant positive association between risk of dementia and adherence to Mediterranean DP (Per each 2 point increase in Mediterranean DP score, HR = 0.92; 95% CI = 0.85, 0.99; P = 0.021).	Age, sex, education, energy intake, smoking status, BMI, waist circumference, household/recreational physical activities, hypertension, hyperlipidaemia, coffee and tea consumption, intake of potatoes, eggs, cakes and biscuits. For women only, menopausal status, oral contraceptive use and HRT use.
Charisis et al., (2021) [45]; *Hellenic LongitudinaI Investigation of Aging and Diet (HELIAD);* Greece.	3.1 ± 0.9	1,046	Community dwelling individuals aged over 64 (40.3% male).	73.1 ± 5	Semi-quantitative, validated FFQ; 1 time point.	1. Mediterranean diet, from Panagiotakos et al. (2007).	Dementia diagnosis confirmed using guidelines from DSM-IV-TR. Subtypes (vascular, lewy body and frontotemporal via respective criteria). Probable or possible AD diagnosis based on NINCDS/ADRDA criteria.	Incident dementia	1. Significant positive association between adherence to Mediterranean DP and likelihood of incident dementia (HR, Q_1 vs Q_4 = 0.10; 95% CI = 0.01, 0.78; P = 0.029).	Age, sex, education, energy intake, BMI, clinical comorbidities, MCI at baseline, evaluation centre, physical activity, ApoE genotype.
De Crom et al., (2022) [119]; *Rotterdam Study*; Netherlands.	15.6	2,861 (baseline II)-5,375 (baseline I)	Data from first two cohorts of Rotterdam study, individuals aged 55 and over (57.4-59% female).	67.7 ± 7.8 (baseline I)	170-item FFQ (baseline I), 389-item FFQ (baseline II); 2 time points.	1. MIND from Morris et al. (2015).<br><br>2. Dutch dietary guidelines, from Voortman et al., (2017).<br><br>3. Mediterranean diet, from Panagiotakos et al. (2007).	Dementia diagnosis made by consensus panel based on criteria for all-cause Dementia (DSM-III-R) and AD (NINCDS-ADRDA).	All-cause dementia	1. Positive association between higher MIND adherence and reduced risk of dementia (HR = 0.79; 95% CI: 0.70, 0.91). Cumulative follow-up from baseline II showed better adherence to MIND associated with reduced dementia risk over 5 and 7 years but reduced with longer follow-up periods.<br><br>2. Positive association between higher adherence to the Dutch guidelines and reduced risk of	Age, sex, education, smoking, physical activity, energy intake, BMI, diabetes, hypercholesterolemia, hypertension.

									dementia (HR = 0.89; 95% CI: 0.78, 1.02).<br><br>3. Positive association between higher Mediterranean DP adherence and reduced risk of dementia (HR = 0.75; 95% CI: 0.66, 0.86).	
Hu et al., (2020) [107]; *ARIC;* USA.	27 (median)	13,630	Community-dwelling individuals aged 45-64 years old and living in selected communities (51-68% female).	54 ± 6	66-item semi-quantitative FFQ; 2 time points.	1. HEI-2015, from Krebs-Smith et al., (2018).<br><br>2. AHEI-2010, from Chiuve et al. (2012).<br><br>3. Mediterranean diet, from Fung et al., (2005).<br><br>4. DASH DP, from Fung et al. (2008).	Dementia diagnosis based on neuropsychological battery, interviews and expert review, interviews, hospitalisation or death code for dementia.	Dementia	1. Positive association between HEI-2015 adherence and reduced risk of dementia (HR, Q_5 vs Q_1 = 0.84; 95% CI = 0.73, 0.96; P = 0.01). Association was attenuated after adjusting for cognitive test z-score during visit 2 (HR = 0.86; 95% CI = 0.74–0.99; P for trend = 0.1).<br><br>2. No significant association between adherence to AHEI-2010 and risk of dementia (HR, Q_5 vs Q_1 = 0.98; 95% CI = 0.86, 1.12; P = 0.2).<br><br>3. No significant association between adherence to Mediterranean diet and risk of dementia (HR, Q_5 vs Q_1 = 0.95; 95% CI = 0.83, 1.08; P = 0.3).<br><br>4. No significant association between adherence to DASH DP and risk of dementia (HR, Q_5 vs Q_1 = 1.02; 95% CI = 0.89, 1.16; P = 0.6).	Age, sex, race-centre, total energy intake, education level, APOE ε4 genotype, smoking status, physical activity, alcohol status, BMI, total cholesterol, systolic blood pressure, anti-hypertensive medications, diabetes, CHD history.
Liu et al., (2021) [99]; *WHI-DM;* USA.	18.7	37,689	Post-menopausal women from WHIMS, aged 50-79 at recruitment (100% female).	61.7 ± 6.7	122-item FFQ; 2 time points.	1. Sugar-based DP (a-posteriori DP via RRR).	Incident AD diagnosis ascertained via self-administered questionnaire.	Incident AD	1. Significant association between increased adherence to sugar DP and greater risk of incident AD (HR, Q_4 vs Q_1 = 1.22; 95% CI = 1.07, 1.40; P = 0.004; P for trend = 0.017).	Age, race/ethnicity, sampling design, daily energy intake, education, health insurance, hormone therapy, smoking status, physical activity, alcohol consumption, diabetes

										and glucose lowering drug use.
Lu et al., (2020) [121]; *Ohsaki Cohort Study;* Japan.	5.0 ± 1.4	3,146	Older individuals aged ≥65 years residing in Ohsaki City (46.4% male).	74.0 ± 5.4	39-item validated FFQ; 2 time points.	1. Japanese diet index (JDI), adapted from Matsuyama et al., (2020).	Incident Dementia; Criteria in long term care insurance system in Japan.	Incident dementia	1. Significant association between risk of incident dementia and greater decreased adherence to JDI (HR = 1.72; 95% CI = 1.13, 2.62), moderate decreased adherence to JDI (HR = 1.10; 95% CI = 0.73, 1.66), moderate increase (HR = 0.82; 95% CI = 0.54, 1.25) and great increase (HR = 0.62; 95% CI = 0.38, 1.02) (P for trend = <0.0001).	Sex, age, education, BMI, smoking status, alcohol consumption, time spent walking, daily energy intake, history of stroke, hypertension, myocardial infarction, diabetes, JDI score and psychological distress.
Nicoli et al., (2021) [94]; *Monzino 80+ Study;* Italy.	3.6	512 (longitudinal only)	Individuals aged 80 or above living in Varese province (68.6% female).	92-93	23-item validated FFQ; 1 time point.	1. Mediterranean diet, authors own.	Dementia diagnosis ascertained using criteria from DSM-IV.	Incident dementia	1. No association between adherence to Mediterranean DP and dementia incidence across follow-up (HR, T_3 vs T_4 = 1.17; 95% CI = 0.82, 1.66; HR, T_5 vs T_9 = 1.20; 95% CI = 0.82, 1.76; P for trend = 0.369).	Age, sex, education, total caloric intake, smoking, alcohol, physical activity, hypertension, COPD, diabetes, lifetime depression, stroke history and transient ischaemic attack history.
Samuelsson et al., (2021) [95]; *Gothernburg H70 Birth Cohort Studies;* Sweden.	12.8 ± 4.5	602	Dementia-free 70 year olds (64% female).	70.6 ± 0.3	Diet history recall method via trained interviewer; 1 time point.	1. Western DP (a-posteriori via RRR)<br><br>2. Healthy DP (a-posteriori via RRR)	Dementia diagnosis ascertained via DSM-III	Incident dementia	1. Significant positive association between better adherence to healthy DP and incident dementia risk among APOE E4 non-carriers (HR = 0.77; 95% CI = 0.61; 0.98; P = 0.03), but no association among carriers (HR = 0.86; 95% CI = 0.63; 1.18; P = 0.35).<br><br>2. Significant negative association between higher adherence to Western DP and incident dementia risk among APOE E4 carriers (HR = 1.37; 95% CI = 1.05, 1.78; P = 0.02), but no association among non-	Sex, birth year, BMI, energy intake, serum cholesterol, hypertension, diabetes, physical activity, education, smoking, five principal components to correct for population stratification.

									carriers (HR = 0.99; 95% CI = 0.81, 1.21; P = 0.92).	
Zhang et al., (2021) [93]; *Effects and Mechanism Investigation of Cholesterol and Oxysterol on Alzheimer's Disease (EMCOA);* China.	2.3	2,339	Multi-centre prospective study of community dwelling individuals aged 50-70 years (46% male).	58.8 ± 4.7	85-item semi-quantitative validated FFQ; 1 time point.	1. 2016 Dietary Guidelines and Food Guide Pagoda for Chinese (CDGI-2018), from Wang et al., (2019).	MoCA used to identify potential MCI with educational cut-offs, suspected cases confirmed by neurologist to establish final diagnosis.	MCI	1. Positive association between adherence to CDGI-2018 and reduced risk of MCI across follow-up (RR = 0.75; 95% CI = 0.59, 0.98; P for trend = 0.006).	Age, sex, education, BMI, smoking, drinking, exercise, hypertension, diabetes mellitus, hyperlipidaemia & coronary heart disease.
Zhu et al., (2022) [90]; *Chinese Longitudinal Healthy Longevity Survey (CLHLS);* China.	10	6,136	Nationally representative sample from 22 Provinces in China (53.7% male).	79.53 ± 9.83	Non-quantitative 16-food group FFQ; ≥1-4 time points.	1. Plant dietary index (PDI) from Satija et al. (2016)<br><br>2. Healthy plant dietary index (hPDI) from Satija et al. (2016).<br><br>3. Unhealthful plant-based diet index (uPDI) from Satija et al. (2016).	Chinese version of MMSE, dichotomised with cut off to identify cognitive impairment (≤24).	Cognitive impairment	1. Positive association between adherence to PDI and odds of developing cognitive impairment across follow-up among those with normal cognition at baseline (OR, Q_4 vs Q_1 = 0.45; 95% CI = 0.39, 0.52; P = <0.001).<br><br>2. Positive association between adherence to hPDI and odds of developing cognitive impairment across follow-up among those with normal cognition at baseline (OR, Q_4 vs Q_1 = 0.61; 95% CI = 0.54, 0.70; P = <0.001).<br><br>3. Negative association between adherence to uPDI and odds of developing cognitive impairment across follow-up among those with normal cognition at baseline (OR, Q_4 vs Q_1 = 2.03; 95% CI = 1.79, 2.31; P = <0.001).	Age, sex, marital status, urban/rural residence, education, occupation before age 60, financial status, social and leisure activity, drinking and smoking status, physical activity, geographical region, BMI, vitamin A/C/E intake, status of five cardiometabolic diseases (hypertension, diabetes, heart disease, cerebrovascular disease, dyslipidaemia).

Vu et al., (2022) [87]; *CHAP, MAP & WHIMS;* USA.	Mixed dependent on cohort.	CHAP = 2,449<br><br>MAP = 725<br><br>WHIMS = 5,308	Samples from CHAP, MAP and WHIMS.	CHAP white = 72.2-74; CHAP black = ; MAP = 80.3-82.3; WHIMS = 69.8-70.3.	CHAP = 139-item FFQ; MAP = 144-item semi-quantitative validated FFQ; WHIMS = Semi-quantitative 3-month FFQ; FFQs conducted at multiple timepoints but analysis used data from one time point	1. MIND, from Morris et al. (2015).	CHAP = Diagnosis ascertained using NINCDS and ADRDA guidelines; MAP = Diagnosis ascertained via same as CHAP; WHIMS = Diagnosis ascertained via DSM V4.	All-cause dementia.	CHAP-white = No association between adherence to MIND DP and risk of all-cause dementia (HR, T_3 vs T_1 = 1.23; 95% CI = 0.47, 3.18; P = 0.68).<br><br>CHAP-black = No association between adherence to MIND DP and risk of all-cause dementia (HR, T_3 vs T_1 = 1.48; 95% CI = 0.51, 4.27; P = 0.47).<br><br>MAP = Positive association between adherence to MIND DP and risk of all-cause dementia (HR, T_3 vs T_1 = 0.63; 95% CI = 0.42, 0.92; P = 0.02).<br><br>WHIMS = Positive association between adherence to MIND DP and risk of all-cause dementia (HR, T_3 vs T_1 = 0.80; 95% CI = 0.72, 0.89; P = <0.0001).	Age, sex, MIND score, genotyping platform, years of education, late-life cognitive activity, global cognition, income, hypertension, diabetes, heart disease, stroke history, smoking, calories, CESD score, Physical activity, BMI. WHIMS also included region, randomisation status and GWAS set<br><br>.

Supplementary Table S6. Overview of intervention studies examining effects of DPs on neurocognitive outcomes.

Authors, year, *study name*, country.	Study characteristics							Outcome measures		Results
	Study design	Intervention duration and follow up period where applicable	Methods of randomisation	Sample size	Population characteristics	Intervention description (including duration of each treatment and control intervention)	Dietary pattern(s) assessed and methods of dietary assessment	Primary outcome(s)	Assessment method for primary outcome(s)	Summary of study findings in relation to primary outcome(s)

Arjmand, Abbas-Zadeh and Eftekhari et al., (2022) [127], Iran.	RCT – Parallel group	3 month intervention period.	Computerised web-based random number table.	N = 37<br><br>Mean age = 48 ± 5.3 years<br><br>100% Female	Middle-aged obese women (40-60 years).	Calorie restricted control diet or calorie restricted MIND diet.<br><br>Intervention: Intervention participants received education and instruction at baseline, provided with 7 day menu to meet MIND diet and aimed to reduce calories by 500kcal. Control participants received general information about healthy food choices.	Calorie restricted MIND DP (without alcohol component).<br><br>Diet assessed via 168-item FFQ assessed using Nutritionist IV software. Adherence to MIND diet assessed via MIND scoring questionnaire and 3 day diet recall.	Cognitive performance using neuropsychological tests.	Forward digit span task (FDST), backward digit span task (BDST), letter number sequencing task (LNST), symbol digit modality task (SDMT), auditory verbal learning test (AVLT), trail making test A and B (TMTA and TMTB), Stroop task.	Significant improvements in cognitive performance for both intervention and control groups. When intervention group was compared to control, there was a significant effect (P = <0.05) of a calorie restricted MIND DP on FDST (95% CI = 1.15, 2.35), BDST (95% CI = 0.01, 0.86), LNST, (+1.37; 95% CI = 0.79, 1.95), SDMT (+ 3.75; 95% CI = 2.43, 5.07), TMTA (95% CI = -9.16, -2.22), AVLT (+ 4.85; 95% CI = 3.3, 6.4). No between-group effects were found for TMTB (95% CI: -6.34, 1.09) or Stroop (95% CI: -23.6, 3.09)
Chlebowski et al., (2020) [128] *Women's Health Initiative Memory Study (WHIMS), USA.*	RCT - Parallel group	8.5 year (median) intervention period.<br>18.7 years (median) cumulative follow-up period (intervention plus post-intervention).	Computerised block.	n = 1,606<br><br>Mean age at screening = 69.9 years<br><br>100% Female	Post-menopausal women aged 50-79 years old and participating in Women's Health Initiative Hormone therapy trials.	Low-fat DP vs usual diet (control).<br><br>Intervention: Low-fat dietary pattern, aiming to reduce dietary fat intake to 20% of energy intake. Increased intake of fruits, vegetables and grains. Delivered by trained certified nutritionists. 18 group meetings in year one and quarterly thereafter during 8.5y intervention period (median).	WHIMS Low-fat DP<br><br>Diet assessed via 4-day food record and FFQs at baseline, 24-hr recalls for post-intervention studies.	Cognitive impairment or probable dementia.	Global cognitive function via 3MSE. Suspected cognitive impairment assessed further using CERAD and assessment via clinical experts. Participants classified into three groups; 1. No cognitive impairment; 2. MCI; 3. Probable dementia (PD).	Significant reduction in risk of possible cognitive impairment (HR = 0.59; 95% CI = 0.38, 0.91, P = 0.01), MCI (HR = 0.65; 95% CI = 0.35, 1.19) and probable dementia (HR = 0.63; 95% CI = 0.19, 2.10) within intervention group.<br>After 18.7 years, no significant difference between intervention and control group in risk of death from all-causes after possible cognitive impairment (HR = 0.83; 95%CI = 0.35, 2.00, P = 0.16).
Knight et al., (2016) [14] *MedLey,* Australia.	RCT - Parallel group	6 months	Minimisation process.	*n* = 137<br><br>Mean age = 72.1 ± 5.0 years<br><br>53.3% Female	Healthy older adults aged ≥65 years old.	Mediterranean diet vs control diet (habitual dietary intake).<br><br>Med Diet Groups received: Met with dietitian, monitored fortnightly to educate and check compliance. Received free food in form of legumes, yoghurt, EVOO, canned tuna, walnuts, peanuts and almonds.<br><br>Control groups: Maintain customary	Adaptation of Mediterranean DP<br><br>Diet assessed via FFQ, 3-d food record and semi-quantitative daily food list.	Cognitive change.	Measured via Executive function (using Stroop test (Dodrill's version), initial letter fluency, excluded letter fluency and D-KEFS tower of London version), memory (using RAVLT, digit span forward and backward and letter number sequencing from WAIS-IV), and processing speed (using symbol search and coding (WAIS-IV), BVRT	No significant differences between intervention vs control group for any domain of cognition (executive function, memory, processing speed, visual-spatial performance or overall age-related cognitive performance.

						lifestyle/diet pattern. Provided with money gift vouchers to local supermarkets.			and visual-spatial memory).	
Kwok et al., (2012) [126], Hong Kong.	RCT - Parallel group	Mean: 25 months<br><br>Range: 24-33 months	Hostels randomised. Two hostels randomised en bloc.	*n* = 429<br><br>Mean age = 83<br><br>84.6% Female	Adults aged ≥75 years old, living in old age hostels.	Intervention to increase intake of fruit, vegetables and fish and decrease intake of salt. Group diet counselling and menu changes provided to intervention group. Control group received advice about hostel menu only.	Healthy DP<br><br>Diet assessed via 24hr recall or food record via interview with trained research assistant for cognitively able individuals.<br><br>For individuals with MCI and dementia, diet assessed via observation at three main meals by research assistant.	Cognitive change.	Measured via appropriate clinical dementia rating scale (CDR), Chinese MMSE and category fluency.	No significant effect on cognition noted in either control or intervention group. However intervention was effective in maintaining consumption of fruit and fish.
Marseglia et al., (2018*) [129], NU-AGE,* Europe.	RCT - Parallel group	1 year intervention	Computer generated allocation	*n* = 1,279<br><br>Mean age = 70.9 ± 3.4<br><br>56.3% Female	Relatively healthy older adults aged 65-79 years old.	Individually tailored Nu-Age diet vs control diet (habitual dietary intake).<br><br>Nu-Age Intervention group:<br>Received diet advice for individual tailoring of Nu-Age food based guidelines. Individual meetings with dietitian/nutritionist via phone and face to face monthly. Included motivational interviewing techniques. Some participants received free food (Italy).<br><br>Control group:<br>Received leaflet featuring advice for national diet guidelines.	NuAge food-based guidelines.<br><br>Diet assessed via 7-d food record for 7 days over 2 weeks following baseline, followed by interview with dietitian/researc h nutritionist to review the record.	Cognitive change.	Measured via global cognition (MMSE and CERAD), perceptual speed (pattern comparisons and digit cancellation), executive function (category fluency and trail making test B/A ratio), episodic memory (World List Memory - immediate and delayed recall and Babcock Story Recall Test - immediate and delayed recall), verbal abilities (15 items boston naming test), constructional praxis (constructional praxis test).	Improvements in global cognition across all groups noted but no significant difference in change between intervention and control groups.<br>Within the intervention group alone, better adherence to Nu-Age DP produced significant improvements in global cognition (β = 0.20; 95% CI = 0.004, 0.39; P = 0.046) and episodic memory (β = 0.15; 95% CI = 0.02, 0.28; P = 0.025) at follow-up compared to those with lower adherence.

Martinez-Lapiscina et al., (2013) [12] *PREDIMED-NAVARRA (arm of PREDIMED)* Spain.	RCT - Multi-arm parallel groups (3)	6.5 year intervention	Computerised random-number sequence.	*n* = 522<br><br>Age = 74.6 ± 5.7<br><br>55.4% Female	Community dwelling males aged 55-80 y and females aged 60-80 y free of CVD initially with high vascular risk.	Mediterranean DP + EVOO and<br>Mediterranean DP + Nuts<br>vs<br>low fat diet group (control).<br><br>Mediterranean DP groups: Individuals received suggestions for lifestyle changes and free supplementation (EVOO for 1L/week or nuts for 30g per day)<br><br>Control groups: Individuals received suggestions to follow a low-fat diet.	Mediterranean DP<br><br>Diet assessed via 137-item FFQ and 14-item questionnaire of adherence to Mediterranean DP during interview with trained dietitian.	Cognitive change.	Measured via MMSE and clock drawing test (CDT).	Significant difference in MMSE (Adjusted difference = 0.62; 95% CI: 0.18, 1.05; P = 0.005) and CDT (Adjusted difference = 0.51; 95% CI: 0.20, 0.82; P = 0.001) within MeDi + EVOO group compared to controls.<br><br>Significant difference in MMSE (Adjusted difference = 0.57; 95% CI: 0.11, 1.03; P = 0.015) and CDT (Adjusted difference = 0.33; 95% CI: 0.003, 0.67; P = 0.0048) within MeDi + Nuts group compared to controls.
Valls-Pedret et al., (2015) [13] *PREDIMED,* Spain.	RCT - Multi-arm groups (3)	4.1 year intervention	Computerised random-number sequence by gender and age.	*n* = 447<br><br>Mean age = 66.9<br><br>52.1% Female	Community dwelling males aged 55-80 y and females aged 60-80 y free of CVD initially with high vascular risk.	Mediterranean DP + EVOO and<br>Mediterranean DP + Nuts<br>vs<br>low fat diet group (control).<br><br>Mediterranean DP groups: Individuals received suggestions for lifestyle changes and free supplementation (EVOO for 1L/week or nuts for 30g per day)<br><br>Control groups: Individuals received suggestions to follow a low-fat diet.	Mediterranean DP.<br><br>Diet assessed via 137-item FFQ and 14-item questionnaire of MeDi adherence during interview with trained dietitian.	Cognitive change.	Measured via MMSE, RAVLT, verbal paired associates and animal fluency tests.	Among control group, cognitive composite scores decreased significantly from baseline (P = <0.05).<br><br>Significant difference in RAVLT (Mean difference = 4.50; 95% CI = 3.24, 5.77; P = 0.049), CDT (Mean difference = 5.66; 95% CI = -10.23, 21.55; P = 0.045), frontal cognition composite score (Mean adjusted difference = 0.23; 95% CI = 0.03, 0.43; P = 0.003) and global cognition composite (Mean adjusted difference = 0.05; 95% CI = -0.11, 0.21; P = 0.005) within MeDi + EVOO group compared to controls.<br><br>Significant difference in composite memory score (Mean adjusted difference = 0.09; 95% CI = -0.05, 0.23; P = 0.04) within MeDi + Nuts group compared to control.

Wade et al., (2018) [123] *MedDairy,* Australia [.	RCT - Parallel group crossover design	24 weeks (8 week intervention/control followed by 8 week washout period, followed by 8 week control/intervention)	Block randomisation stratified by age and gender.	*n*= 41<br><br>Mean age = 60.2 ± 6.9<br><br>68.3% Female	Adults aged 45-80 with cardiovascular risk factors.	Mediterranean DP supplemented with dairy (2-3 servings daily of dairy foods) vs low-fat diet (control).<br><br>MedDairy group: Advised to follow Mediterranean DP while consuming dairy foods 2-3 times per day. Free food provided (greek yoghurt, almonds, walnuts, hazelnuts, EVOO, cheese slides, peas, beans, chickpeas, lentils and tuna). Attended bi-weekly visits with dietitan to discuss adherence/challenges and adverse effects.<br><br>Control groups: Instructed to follow habitual diet but reduce total fat consumption.<br><br>Both groups received dietetic counselling, meeting with dietitian at beginning of intervention for education guidelines and resources.	Mediterranean DP enriched with dairy (MedDairy).<br><br>Diet assessed via 3-day weighed food records. DP scored using Trichopoulou et al., (Trichopoulou et al. 2003) and Sofi et al., (reversed dairy scoring).	Cognitive change.	Measured via CANTAB for memory, processing speed and planning.	Significant effect of MedDairy diet on processing speed at 8 weeks compared to control (LF diet) (Estimated mean difference between interventions = 0.185; 95% CI = 0.008, 0.361; P = 0.04). No other significant effects from intervention compared to control in domain of attention, memory, planning or on ACE-R.
Wade et al., (2019) [124] *MedPork,* Australia.	RCT - Parallel group crossover design	24 weeks (8 week intervention/control followed by 8 week washout period, followed by 8 week control/intervention)	Block randomisation stratified by age and gender.	*n* = 35<br><br>Mean age = 61 ± 7.1<br><br>69.7% Female	Adults aged 45-80 with cardiovascular risk factors.	Mediterranean DP supplemented with pork (2-3 servings weekly of fresh lean pork) vs low-fat diet (control).<br><br>MedPork group: Advised to follow Mediterranean DP while consuming fresh lean pork 2-3 times per week (provided free). Attended bi-weekly visits with dietitan to discuss adherence/challenges and adverse effects.<br><br>Control groups:	Mediterranean DP enriched with pork (MedPork)<br><br>Diet assessed via 3-day weighed food records. DP scored using Trichopoulou et al., and Sofi et al., (reversed meat scoring).	Cognitive change.	Measured via CANTAB for memory, processing speed and planning. Used ACE-R to exclude individuals with MCI or dementia at week 8 and 24.	Significant effect of MedPork diet on processing speed at 8 weeks compared to control (LF diet) (Estimated mean difference = 0.32; 95% CI = 0.08, 0.57; P = 0.01). No other significant effects from intervention compared to control in domain of attention, memory, planning or on ACE-R.

						Instructed to follow habitual diet but reduce total fat consumption.<br><br>Both groups received dietetic counselling, meeting with dietitian at beginning of intervention for education guidelines and resources.				
Nilsson et al., (2013) [125], Sweden	RCT - Parallel group crossover design	4 weeks intervention period, 4 weeks washout, 4 weeks control		*n* = 44<br><br>Mean age = 63.3 ± 0.8<br><br>82% Female	Healthy individuals (without any medical condition or cognitive decline) aged 50-73.	Intervention using diet to reduce inflammation/cardiometabolic risk vs control (similar distribution of energy but lacked active food components/concepts). Each diet followed for 4 week period followed by washout period of 4 weeks in between.<br><br>Intervention group: Received 14-day menu plan repeated during last two weeks. Recipes provided for each meal including details on cooking methods and quantities.	DP to reduce inflammation/cardiometabolic risk based on Nordic Recommendations.<br><br>Diet assessed via checklist to tick off active food components and noted deviations from meal plan.	Cognitive function.	Measured via selective attention (SA-test) and learning and memory (RAVLT).	Intervention diet found to improve performance on RAVLT recognition test (P = < 0.05) and selective attention test.

Supplementary Table S7. Results of risk of bias assessment for intervention studies using RoB2.

Study	Risk of bias domains					Overall bias
	Random-isation	Deviation	Missing data	Measurement	Selection	
**Arjmand et al., (2022) [127]**	Low	Some concerns	Some concerns	Low	Low	**Some concerns**
**Chlebowski et al., (2020) [128]**	Low	Some concerns	High	High	Some concerns	**High**
**Knight et al., (2016) [14]**	Low	Some concerns	Low	Low	Low	**Some concerns**
**Kwok et al., (2012) [126]**	High	Some concerns	Low	Some concerns	Low	**High**
**Marseglia et al., (2018) [129]**	Low	Some concerns	Low	Some concerns	Low	**Some concerns**
**Martinez-Lapiscina et al., (2013) [12]**	Low	Some concerns	High	Some concerns	Some concerns	**High**
**Nilsson et al., (2013) [125]**	High	Some concerns	Some concerns	High	Some concerns	**High**
**Valls-Pedret et al., (2015) [13]**	Low	Some concerns	High	Some concerns	Some concerns	**High**
**Wade et al., (2018) [123]**	Low	Some concerns	Some concerns	High	Low	**High**
**Wade et al., (2019) [124]**	Some concerns	Some concerns	Some concerns	High	Low	**High**
